# The relationship between cytokine and neutrophil gene network distinguishes SARS-CoV-2–infected patients by sex and age

**DOI:** 10.1172/jci.insight.147535

**Published:** 2021-05-24

**Authors:** Paula P. Freire, Alexandre H.C. Marques, Gabriela C. Baiocchi, Lena F. Schimke, Dennyson L.M. Fonseca, Ranieri C. Salgado, Igor S. Filgueiras, Sarah M.S. Napoleao, Desirée R. Plaça, Karen T. Akashi, Thiago Dominguez Crespo Hirata, Nadia El Khawanky, Lasse M. Giil, Gustavo Cabral-Miranda, Robson F. Carvalho, Luis Carlos S. Ferreira, Antonio Condino-Neto, Helder I. Nakaya, Igor Jurisica, Hans D. Ochs, Niels Olsen Saraiva Camara, Vera Lúcia G. Calich, Otavio Cabral-Marques

**Affiliations:** 1 Department of Immunology, Institute of Biomedical Sciences, and; 2 Department of Clinical and Toxicological Analyses, School of Pharmaceutical Sciences, University of São Paulo, São Paulo, Brazil.; 3 Department of Hematology and Oncology, Faculty of Medicine, University of Freiburg, Freiburg, Germany.; 4 Department of Internal Medicine, Haraldsplass Deaconess Hospital, Bergen, Norway.; 5 Department of Structural and Functional Biology, Institute of Biosciences, São Paulo State University, Botucatu, São Paulo.; 6 Vaccine Development Laboratory, Institute of Biomedical Sciences, Department of Microbiology, University of São Paulo, São Paulo, Brazil.; 7 Krembil Research Institute, University Health Network, and Departments of Medical Biophysics and Computer Science, University of Toronto, Toronto, Canada.; 8 Department of Pediatrics, University of Washington School of Medicine, and Seattle Children’s Research Institute, Seattle, Washington.; 9 Network of Immunity in Infection, Malignancy, and Autoimmunity, Universal Scientific Education and Research Network, São Paulo, Brazil.

**Keywords:** COVID-19, Inflammation, Cytokines, Molecular genetics, Neutrophils

## Abstract

The fact that the COVID-19 fatality rate varies by sex and age is poorly understood. Notably, the outcome of SARS-CoV-2 infections mostly depends on the control of cytokine storm and the increasingly recognized pathological role of uncontrolled neutrophil activation. Here, we used an integrative approach with publicly available RNA-Seq data sets of nasopharyngeal swabs and peripheral blood leukocytes from patients with SARS-CoV-2, according to sex and age. Female and young patients infected by SARS-CoV-2 exhibited a larger number of differentially expressed genes (DEGs) compared with male and elderly patients, indicating a stronger immune modulation. Among them, we found an association between upregulated cytokine/chemokine- and downregulated neutrophil-related DEGs. This was correlated with a closer relationship between female and young subjects, while the relationship between male and elderly patients was closer still. The association between these cytokine/chemokines and neutrophil DEGs is marked by a strongly correlated interferome network. Here, female patients exhibited reduced transcriptional levels of key proinflammatory/neutrophil-related genes, such as CXCL8 receptors (*CXCR1* and *CXCR2*), *IL-1β*, *S100A9*, *ITGAM*, and *DBNL*, compared with male patients. These genes are well known to be protective against inflammatory damage. Therefore, our work suggests specific immune-regulatory pathways associated with sex and age of patients infected with SARS-CoV-2 and provides a possible association between inverse modulation of cytokine/chemokine and neutrophil transcriptional signatures.

## Graphical abstract

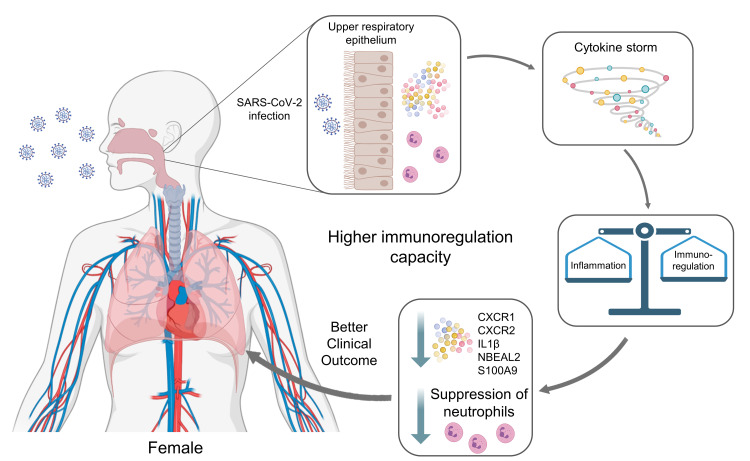

## Introduction

More than 15 months after the outbreak of the novel COVID-19 in Wuhan, China (1–3), approximately 122 million confirmed cases of COVID-19 and more than 2.7 million deaths have been reported worldwide (4). The clinical spectrum of COVID-19 ranges from asymptomatic to severe pulmonary disease, leading to acute respiratory distress syndrome (5, 6). Enhanced expression of the angiotensin-converting enzyme 2 (ACE2), the SARS-CoV-2 entry receptor (7), and dysregulation of the immune response likely contribute to more severe disease in older patients with comorbidities. Even with an increased understanding of what enhances the risk of severe disease, the high male/female mortality ratio remains poorly understood, especially considering the lack of sex differences in disease incidence (6, 8). Immunologically, a recent study reported no sex differences in the levels of anti–S1-IgM and -IgG antibody titers nor the number of naive or memory B cells (9). Compared with male subjects, peripheral blood mononuclear cells from female subjects exhibited higher levels of terminally differentiated T cells expressing activation molecules (CD38 and HLA-DR) and negative regulators (PD-1 and TIM-3) (10). Moreover, male patients displayed higher plasma levels of proinflammatory cytokine/chemokines (*CXCL8* and *IL-18*) (9). These findings indicate a better capacity for immune modulation in female subjects compared with male subjects.

The immune response to SARS-CoV-2 is characterized by hyperactivated T cells (both CD4^+^ and CD8^+^) (6, 11) and macrophages (12). These hyperactivated immune cells likely contribute to the massive serum levels of proinflammatory cytokines, also referred to as “cytokine storm” (13). In parallel, tissue damage has been increasingly associated with neutrophil hyperactivation. This involves neutrophil-induced oxidative stress and high neutrophil counts (14), degranulation, and the release of extracellular traps (NETs). These processes are associated with increased levels of acute-phase reactants (e.g., C-reactive protein), microvascular damage, arterial thrombosis, and red blood cell dysfunction (14, 15). Interestingly, after the first steps of neutrophil hyperactivation in the combat of SARS-CoV-2, the host immune system proportionally increases the production of granulocytic myeloid-derived suppressor cells (MDSCs) when the severity of COVID-19 advances (16–18). Besides their known roles in tumor immune evasion, MDSCs are physiological components of the healthy immune system that expand during life-threatening autoimmune and inflammatory diseases (19, 20), traumatic stress, transplantation, and sepsis (21–23). Thus, the suppression of key neutrophil effector functions can represent a poorly investigated pathophysiological attempt to protect the host against tissue damage in patients with COVID-19.

Several omic studies have been conducted in patients with COVID-19 to help elucidate the molecular mechanisms underlying the disease. Lieberman et al. (24) and Mick et al. (25) recently characterized transcriptional profiles of nasopharyngeal swabs from 668 individuals with SARS-CoV-2 (SC2) and 157 SC2-negative individuals (Neg. SC2). Although these studies have highlighted differences in immune responses that underlie disparities in male and elderly outcomes, an integrative transcriptomic approach investigating the association between cytokines and neutrophil-mediated immunity (NMI) is still missing. The specific set of immune system genes in female subjects underlying the protective mechanisms requires further understanding. Here, we characterize what we believe to be a previously unnoticed interconnected transcriptome network between differentially expressed genes (DEGs) in publicly available transcriptome data sets of nasopharyngeal swabs and peripheral blood leukocytes of human samples (Supplemental Table 1; supplemental material available online with this article; https://doi.org/10.1172/jci.insight.147535DS1). In this context, we focused on the association between the upregulated cytokine-mediated signaling pathway (CMSP) and downregulation of NMI genes according to sex and age. The network of upregulated NMI genes and its association with COVID-19 development will be published elsewhere.

## Results

### Association between cytokine/chemokine- and neutrophil-related genes in nasopharyngeal swabs.

**Figure 1:**
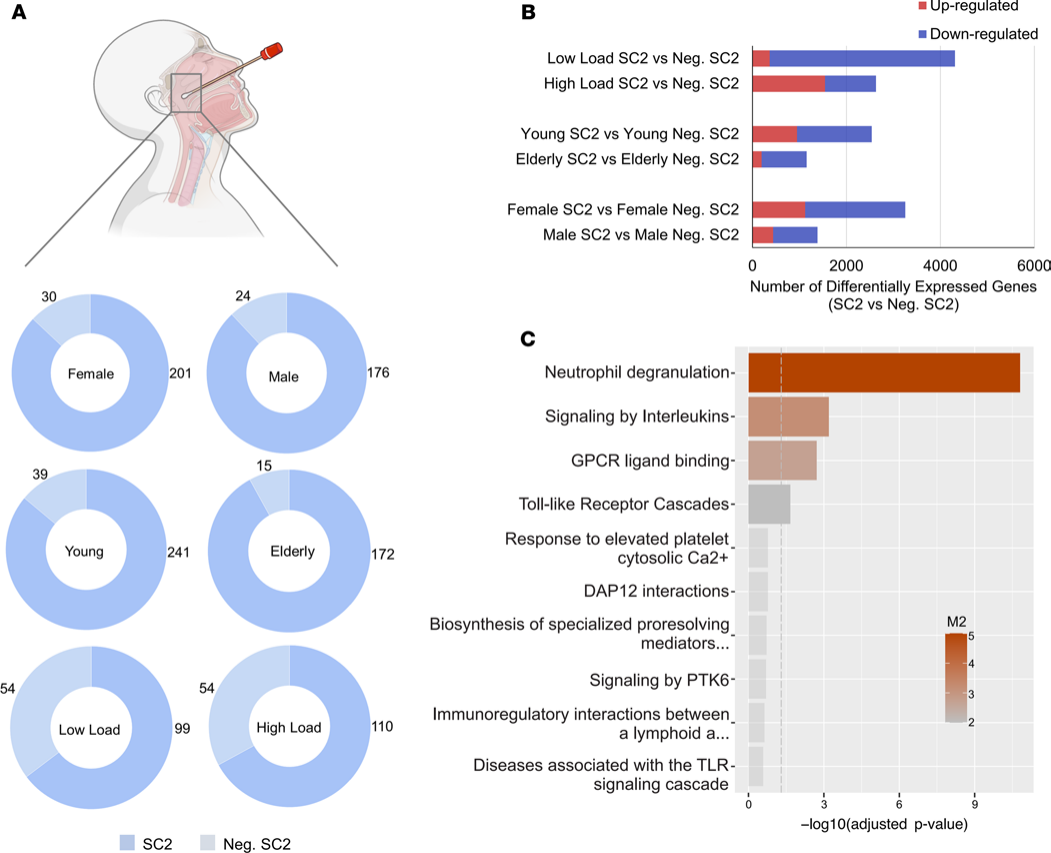
Transcriptomic analysis of swabs from SARS-CoV-2–positive compared with SARS-CoV-2–negative patients (data set GSE152075). (**A**) Number of SARS-CoV-2–positive (SC2) and –negative samples by groups: sex, age (young, <60 years old; elderly, ≥60 years old), and viral load (low and high). (**B**) Total number of differentially expressed genes in SC2-positive samples by group. (**C**) Functional overrepresentation obtained by modular gene coexpression analysis (see Supplemental Figure 1). SC2, SARS-CoV-2 group; Neg. SC2, negative SARS-CoV-2 group.

We first reanalyzed transcriptomic data of nasopharyngeal swabs from patients described by Lieberman et al. (GSE152075) (24) to characterize the expression landscape of immune system genes. We divided the samples according to sex, age (elderly, >60 years old, and young, <60 years old), and viral load (413 patients infected with SARS-CoV-2 and 54 negative controls) (Figure 1A). Groups of patients with high and low viral loads, and younger and older age, were sex matched to avoid confounding effects (Supplemental Tables 1 and 2). Female and young patients infected by SARS-CoV-2 exhibited a higher number of total (up- and downregulated) DEGs when compared with male and elderly patients (Figure 1B and Supplemental Table 3). Genes significantly deregulated in each group of patients were selected to perform enrichment analysis using the Gene Ontology (GO) terms through the Enrichr tool (26, 27). Overall, we found CMSP and neutrophil-mediated immunity (NMI) categories enriched by up- and downregulated genes, respectively, among the compiled top-10 biological process categories of each group (Supplemental Tables 4 and 5).

**Figure 2:**
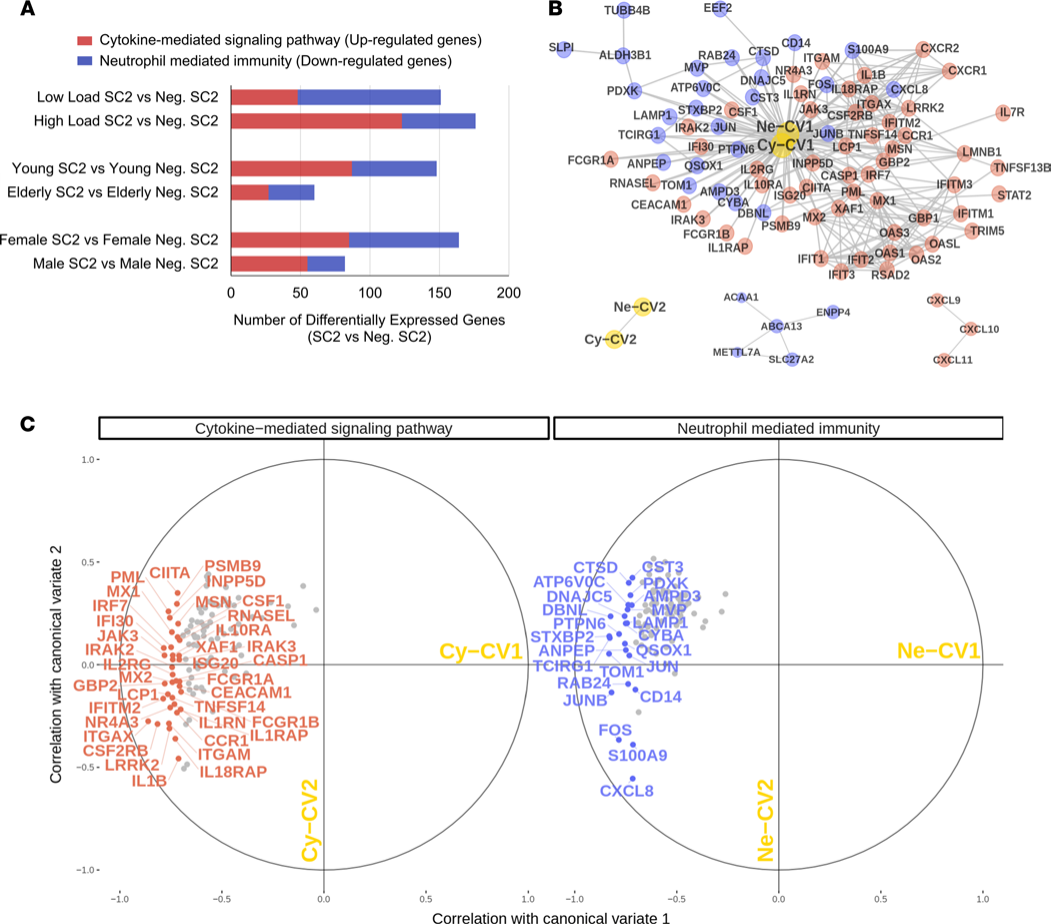
Strength of the association between the upregulated cytokine genes and downregulated neutrophil genes. (**A**) Total number of DEGs associated with cytokine-mediated signaling pathways (CMSP, red bars) and neutrophil-mediated immunity (NMI, blue bars) when comparing SC2-positive patients with viral load-, sex-, and age-matched negative SC2 samples. (**B**) Network representation of matrix of Pearson correlations between genes, including also the estimated canonical variables. Gray edges connect pairs of genes with a Pearson correlation of ≥0.7, and those with a correlation of <0.7 were omitted. (**C**) Heliographic representation of the canonical-correlation analysis between CMSP and NMI genes, showing correlation of genes with their corresponding canonical variates, Cy-CV1 and Cy-CV2 and Ne-CV1 and Ne-CV2, respectively. CMSP and NMI genes with a correlation of ≥0.7 are colored in red and blue, respectively, while those with a correlation of <0.7 are gray in both groups. SC2, SARS-CoV-2 group; Neg. SC2, negative SARS-CoV-2 group; Cy-CV1, canonical variable 1 associated with CMSP genes; Cy-CV2, canonical variable 2 associated with CMSP genes; Ne-CV1, canonical variable 1 associated with NMI genes; Ne-CV2, canonical variable 2 associated with NMI genes.

We used CEMiTool (28) to gain insights into the systemic function of nasopharyngeal swab genes by performing modular coexpression enrichment and network analyses. Among the gene modules identified, module 1 (M1) indicated a coexpression link between genes associated with neutrophil degranulation signaling by ILs and GPCR ligand binding (chemokines and their receptors) (Figure 1C). When we compared the total number of genes associated with CMSP and NMI categories, female and young patients presented higher numbers of up- and downregulated DEGs associated with these categories, respectively, compared with male and elderly patients (Figure 2A and Supplemental Tables 6 and 7). Female patients displayed an enhanced quantity of DEGs compared with male patients, elderly patients, and patients with low viral load (described in Supplemental Tables 6 and 7). These observations indicate a higher transcriptional modulation capacity of the immune response in female and young patients compared with male and elderly patients. Supplemental Tables 8 and 9 contain the complete list of all genes belonging to CMSP and NMI categories, respectively.

Next, we evaluated the strength of the association between the upregulated CMSP and downregulated NMI genes (described in Supplemental Tables 6 and 7). We used canonical-correlation analysis (CCA), which is a multivariate statistical model used to quantify relationships between two groups of interrelated and interdependent variables (29). On each group, CCA finds linear combinations of the corresponding observed variables, forming pairs of linear combinations that are maximally correlated. From the CCA of the aforementioned CMSP and NMI genes, further analysis was performed only on the first 2 pairs of linear combinations, which we named Ne-CV1/Cy-CV1 and Ne-CV2/Cy-CV2 (Figure 2, B and C). Only the pair Ne-CV1/Cy-CV1 was statistically associated, indicating an important connection between CMSP and NMI networks in the immune response to SARS-CoV-2. Among others, the CCA showed an association among transcripts of IL-1β, IL-18 receptor accessory protein (*IL-18RAP*), C-C chemokine receptor type 1 (*CCR1*), IFN-induced guanylate-binding protein 2 (*GBP2*), and IFN regulatory factor (*IRF7*) with NMI transcripts such as C-X-C motif chemokine ligand 8 (*CXCL8*, also called IL-8), lysosome-associated membrane protein 1 (*LAMP1*), and cytochrome B-245 α chain (*CYBA*). Among others, the association of these transcripts suggests an orchestrated modulation among IFN-regulated genes (*GBP2*, *IRF7*, and *IFI30*), neutrophil recruitment degranulation (*CXCL8* and *LAMP1*), and oxidative stress (*CYBA*). The CCA analysis suggests that these 2 sets (CMSP and NMI) of highly correlated genes have a systemic representativity, suggesting pathophysiological relevance in patients with COVID-19.

### CMSP and NMI modular gene coexpression and enrichment in whole blood leukocytes from patients with COVID-19.

**Figure 3:**
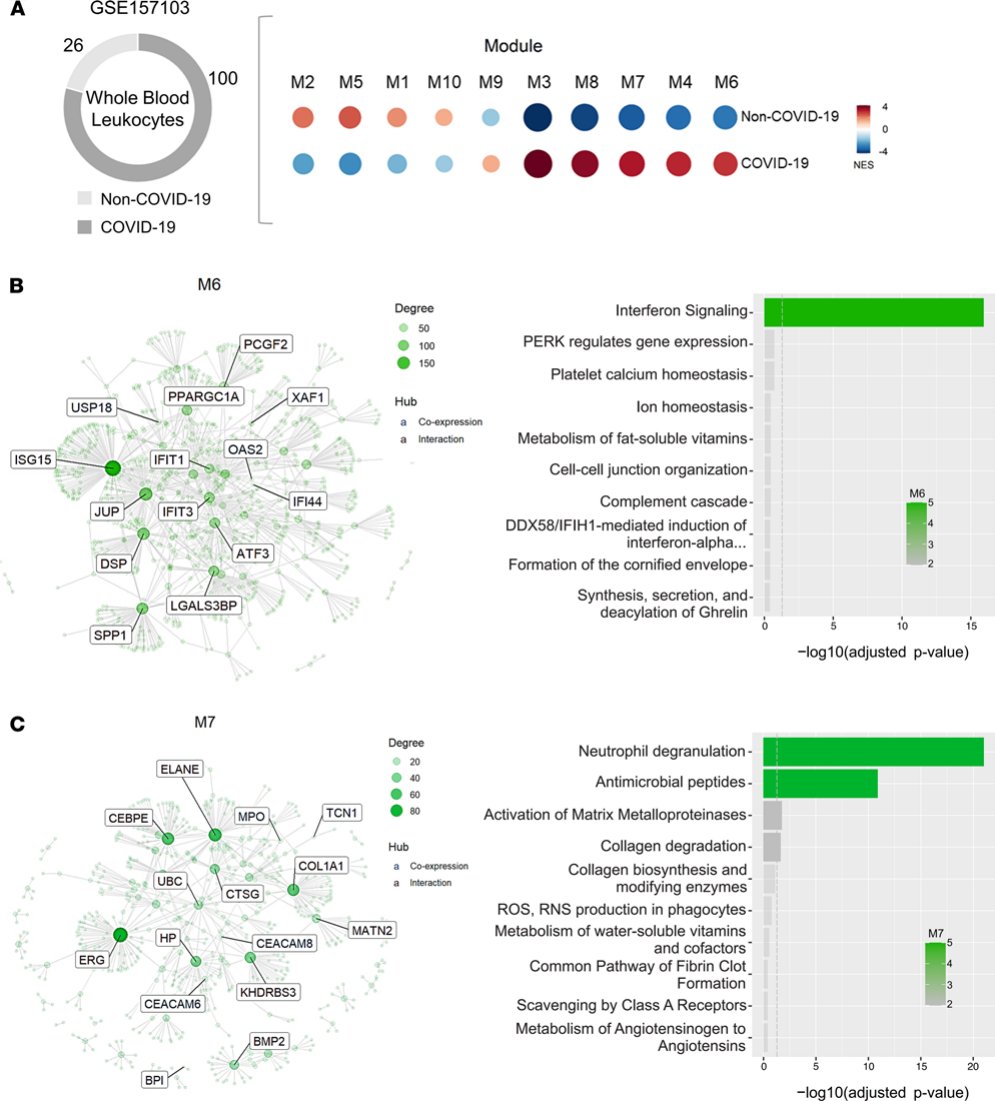
Modular gene coexpression analysis of peripheral blood leukocytes from patients with SARS-CoV-2 (data set GSE157103). (**A**) Bubble heatmap showing the gene set enrichment of each module activity in peripheral blood leukocytes (PBLs) from subjects with or without COVID-19 by sex, age (young, <60 years old; elderly, ≥60 years old), and severity (admitted or not at intensive care unit [ICU]). Symbol size and color reflect the normalized enrichment score, as determined by the CEMiTool. (**B** and **C**) Plot of overrepresentation analysis and gene network of coexpression modules showing the enrichment of IFN signaling (module M6; **B**) and the association between neutrophil degranulation and signaling by ILs (module M7; **C**) in PBLs. Gene nodes are shown in each network, with potential hubs demonstrated inside rectangles; each node size is proportional to its degree of interactivity. NES, normalized enrichment score; M, Module.

Modular coexpression enrichment analyses of whole blood leukocytes from patients with COVID-19 from a recent publicly available data set (GSE157103) (30) showed coherent results compared with the analysis of swabs. CEMiTool analysis revealed 10 coexpression modules enriched in whole leukocytes from patients with COVID-19 compared with the control group. Among them, module M6 was enriched by IFN signaling genes, while module M7 was composed of genes associated with neutrophil degranulation in the COVID-19 group (Figure 3). The data, therefore, point toward a systemic (not restricted to upper airways) immunopathological association between IFN signaling and NMI in patients with COVID-19. Supplemental Tables 10 and 11 describe the DEGs associated with CMSP and NMI in the GSE157103 data set.

### Effect of sex and age on CMSPs in patients with COVID-19.

**Figure 4:**
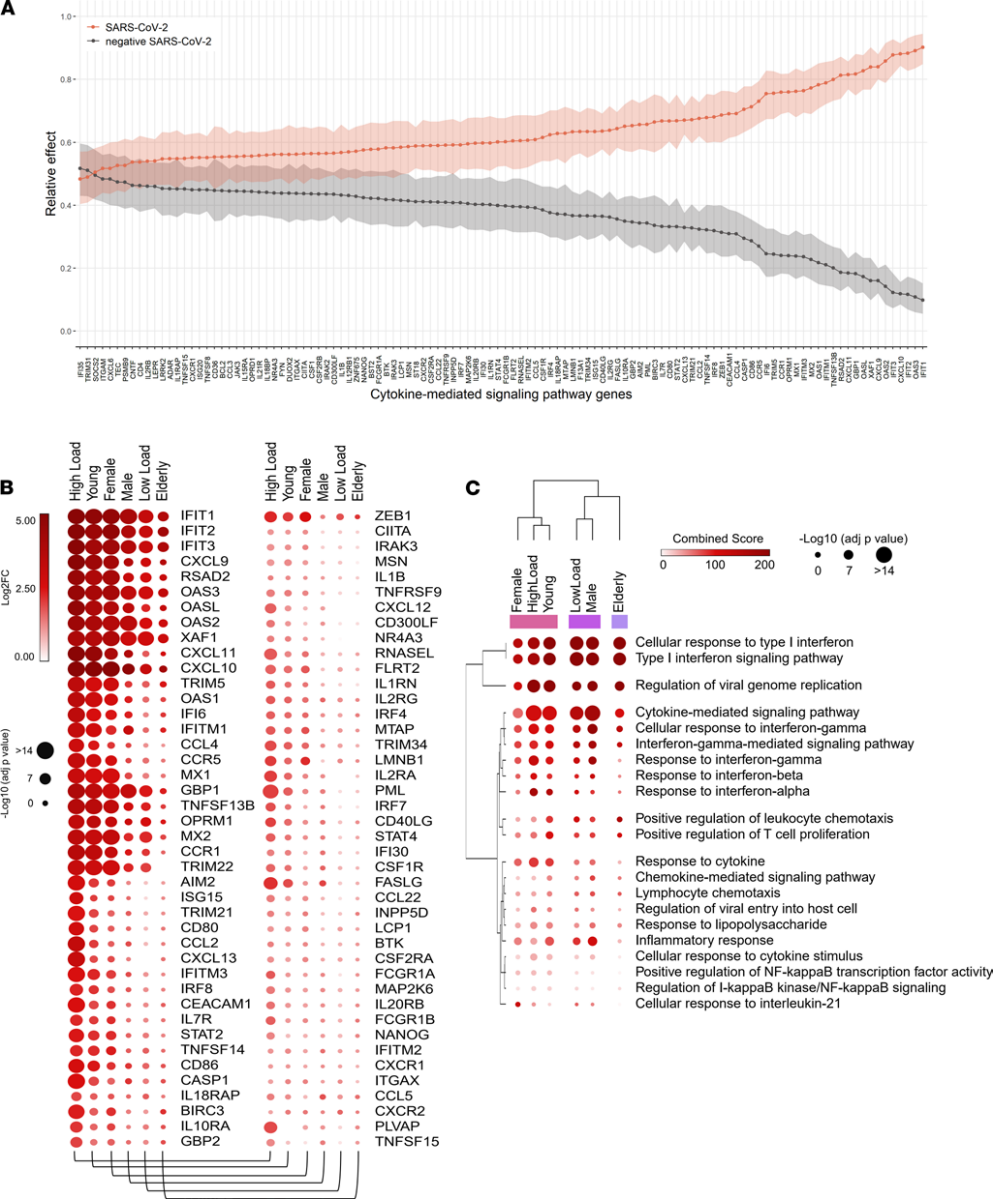
Signature of cytokine-mediated signaling pathway according to sex, age, and viral load. (**A**) Relative effects of cytokine-mediated signaling pathway (CMSP) genes between SARS-CoV-2–positive (SC2) and –negative (Neg. SC2) groups. (**B**) Bubble heatmap showing the expression pattern of CMSP genes by viral load, sex, and age groups. The size and color of circles correspond to –log_10_-transformed adjusted *P* value and log_2_ fold change (log_2_FC), respectively. The cut-off for upregulated genes was log_2_FC >1 and adjusted *P* < 0.05. Rows and columns were clustered based on Euclidean distance between log_2_FC values. (**C**) Bubble heatmap representing the top-ranked combined scores for biological process associated with CMSP genes. The circles’ size and color correspond to –log_10_-transformed adjusted *P* value and combined score, respectively. Rows and columns were clustered based on Euclidean distance between combined score values. Official gene names are shown in Supplemental Table 13.

To better understand the effect of sex and age on the anti–SARS-CoV-2 immune response in patients with COVID-19, we sought to further characterize the influence of sex and age on the upregulation of CMSP (detailed pro- or antiinflammatory function of each gene is described in Supplemental Table 12). Nonparametric multivariate analysis of variance (NP-MANOVA) and analysis of relative effects (31) for genes associated with CMSP, identified in the nasopharyngeal swabs (GSE152075), revealed a significant difference (NP-MANOVA test statistic = 19.885, *P* < 0.01) between SC2 and Neg. SC2 groups, with relative effects showing an overall upregulated spectrum of gene expression for the SC2 group (Figure 4A). Descriptive analysis of the central tendency and variability (interquartile range) of CMSP transcriptional levels demonstrated the overall tendency in each of the SARS-CoV-2–positive and –negative subgroups (female, male, young, elderly, and high and low viral load), corroborating the aforementioned upregulation of CMSP genes in the SARS-CoV-2–positive subgroups (Supplemental Figure 1). Hierarchical clustering analyses of gene expression revealed similarities between the CMSP signature of female and young groups and a close relationship between male and elderly patients. Female and young patients clustered with patients who had high viral loads, the latter presenting the patients with the most upregulated CMSP (Figure 4, B and C). Conversely, male and elderly patients clustered near patients with low viral load. In this context, antiviral transcripts induced by IFNs (32) were most upregulated in female, young, and high-viral-load patients compared with male, elderly, and low-viral-load patients. These included transcripts of IFN-induced protein with tetratricopeptide repeats 1 (*IFIT1*), *IFIT2*, and *IFIT3*; chemokine (C-X-C motif) ligand 9 (*CXCL9*), *CXCL10*, and *CXCL11*; 2′, 5′-oligoadenylate synthetase 1 (*OAS1*), *OAS2*, and *OAS3*; and tripartite motif 5 (*TRIM5*) and *TRIM22*. The same phenomenon was observed in terms of transcripts of endogenous activators of the immune system, such as the CD40 ligand and costimulatory molecules (*CD80* and *CD86*). Thus, our data suggest a higher transcriptional modulation of the immune response triggered by SARS-CoV-2 in female and young patients compared with male and elderly patients.

### Sex and age differences in NMI.

**Figure 5:**
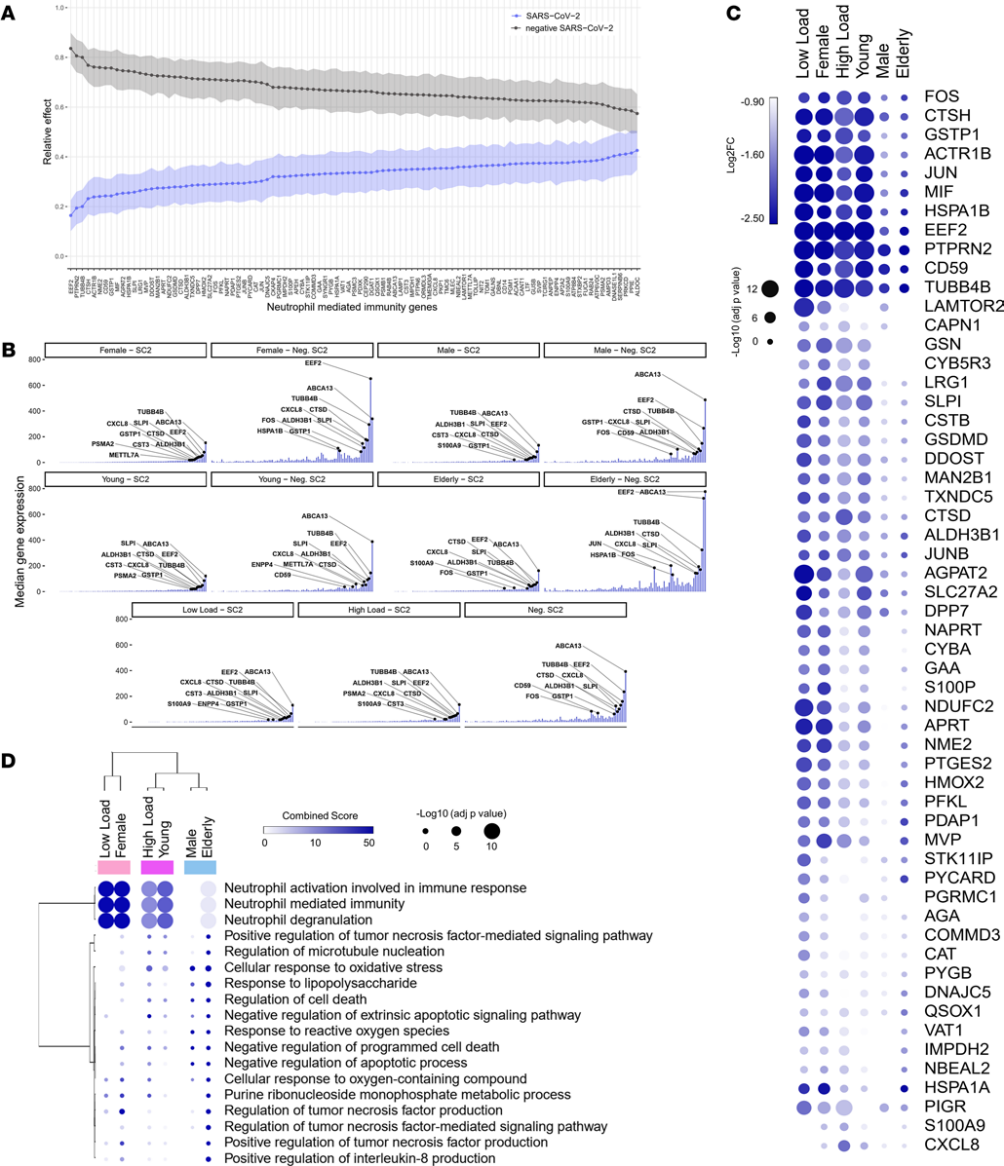
Signature of neutrophil-mediated immunity according to sex, age, and viral load. (**A**) Relative effects of neutrophil-mediated immunity (NMI) genes between SARS-CoV-2–positive (SC2) and –negative (Neg. SC2) groups. (**B**) Central tendency (median) of gene expression for NMI genes in each category versus the viral load-, sex-, and age-matched Neg. SC2 controls. (**C**) Bubble heatmap showing the expression pattern of NMI genes by viral load, sex, and age groups. The size and color of circles correspond to –log_10_-transformed adjusted *P* value and log_2_ fold change (log_2_FC), respectively. The cut-off for upregulated genes was log_2_FC >1 and adjusted *P* < 0.05. Rows and columns were clustered based on Euclidean distance between log_2_FC values. (**D**) Bubble heatmap representing the top-ranked combined scores for biological process associated with NMI genes. The circles’ size and color correspond to –log_10_-transformed adjusted *P* value and combined score, respectively. Rows and columns were clustered based on Euclidean distance between combined score values. Official gene names are shown in Supplemental Table 14.

Similar to CMSP, NP-MANOVA and analysis of relative effects for NMI genes revealed significant differences between SARS-CoV-2–positive and –negative groups (MANOVA test statistic = 19.639, *P* < 0.01). Patients with SARS-CoV-2 infections have been reported to have a higher number of MDSCs (16–18). In agreement with Schulte-Schrepping et al. (16), our differential expression analysis revealed several downregulated genes related to NMI that are associated with MDSCs. These include *CYBA*, *CXCL8*, *CSTB*, *JUN*, *FOS*, and *MIF* (16) (Figure 5, A and B), which encode proteins involved in neutrophil degranulation and activation of oxidative stress and antimicrobial peptides in SARS-CoV-2–positive individuals. The detailed function of each gene is described in Supplemental Table 13. Among them are cathepsins (*CTSB*, *CTSD*, and *CTSH*), migration inhibitory factor (*MIF*), heat shock proteins (heat shock 70 kDa protein 1A [*HSPA1A*] and *HSPA1B*), and cytochrome B-245 α chain (*CYBA*). Hierarchical clustering analysis of NMI genes demonstrated that low viral load, female, and young patients presented a more downregulated transcriptomic profile (Figure 5C). This was seen to a lesser extent in male and elderly patients. The cluster analysis suggests that these expression patterns reflect a close functional relationship (Figure 5D) among low viral load, female sex, and young age in patients.

**Figure 6:**
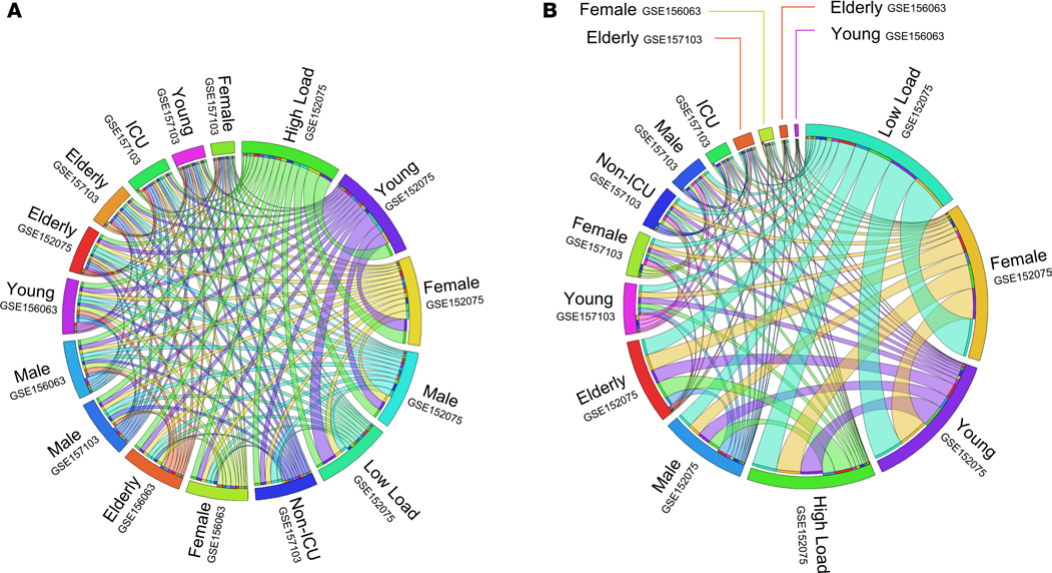
Number of shared cytokine and neutrophil genes among different groups with COVID-19. The thickness of each link (edge) in the Circos plot represents the number of the shared cytokine-mediated signaling pathway (CMSP) (**A**) and neutrophil-mediated immunity (NMI) (**B**) genes throughout the data sets (GSE152075, GSE157103, and GSE156063). Circos plots are distributed by sex, age (young, <60 years old; elderly, ≥60 years old), viral load (low and high), and severity (admitted or not at intensive care unit [ICU]). SC2, SARS-CoV-2 group; Neg. SC2, negative SARS-CoV-2 group; FC, fold change.

We next analyzed the CMSP and NMI genes in 3 different data sets (swabs from GSE152075 and GSE156063 and leukocytes from GSE157103) (24, 25, 30). In Figure 6A, the Circos plot shows that patients with a high viral load shared a higher number of CMSP genes with younger and female patients. Interestingly, female, young, and low viral load patients shared more common NMI genes in swab and total leukocytes than male and elderly patients (Figure 6B). This result suggests a predominant downregulation of NMI genes in groups of patients who do not develop severe disease. Supplemental Figure 2 shows the result of ridge regression analysis, which indicates associations of each gene belonging to the CMSP and NMI group (described below) with viral load, in line with the results presented in Figures 4 and 5. Of note, several genes present in the GSE152075 data set were also identified in another study of nasopharyngeal swabs developed by Mick et al. (25) (Supplemental Figure 3, data set GSE156063), increasing the validity of these findings. Further, we also found a differential expression pattern of CMSP and NMI genes in the bulk and single-cell data sets described above (Supplemental Figure 4).

### Specific immune response–associated gene modulations in female subjects infected with SARS-CoV-2.

**Figure 7:**
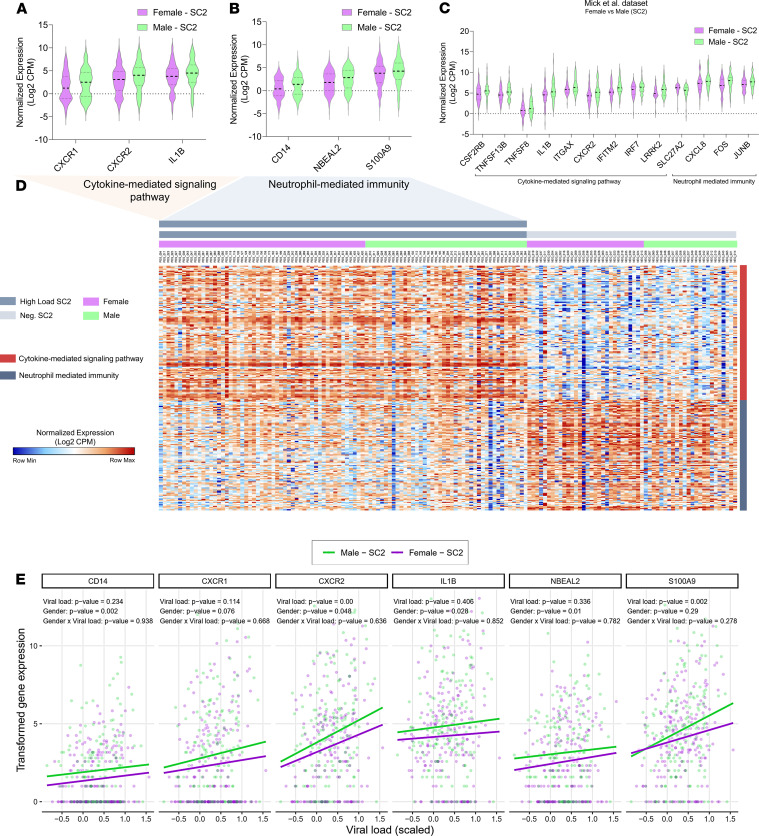
Transcriptional differences in cytokine-mediated signaling pathway and neutrophil-mediated immunity distinguish females and males. (**A** and **B**) Violin plots present differentially expressed genes (DEGs; adjusted *P* < 0.05 and log_2_FC fold change [log_2_FC] >1) of cytokine-mediated signaling pathway (CMSP) (**A**) and neutrophil-mediated immunity (NMI) (**B**) of SARS-CoV-2–positive (SC2) females versus SC2 males from the GSE152075 data set. (**C**) DEGs of swabs from female versus male positive SC2 patients from the GSE156063 data set associated with CMSP and NMI. (**D**) Unsupervised hierarchical clustering heatmap of CMSP and NMI genes (normalized gene expression in log_2_ CPM). Red tracks denote CMSP genes, and blue tracks represent NMI genes. (**E**) Multivariate regression of gene expression of *CD14*, *CXCR1*, *CXCR2*, *IL-1β*, *NBEAL2*, and *S100A9* according to viral load and sex. Results indicate that the variation observed for expression values of these 6 genes dependent mainly on sex-specific differences. SC2, SARS-CoV-2 group; Neg. SC2, negative SARS-CoV-2 group; CPM, counts per million.

Considering the increased male/female mortality ratio (Supplemental Figure 5), we sought to further characterize the transcriptomic features of COVID-19 in a sex-specific manner. We searched for sex differences between DEGs identified in female versus male patients with COVID-19 as well as female versus healthy male controls throughout the different RNA-Seq data sets from Lieberman et al. (24) and Mick et al. (25). We performed differential expression analysis focused on the list of CMSP and NMI genes according to GO functional annotation (http://geneontology.org/). In the Liberman data set (24), 6 genes showed a statistical difference between male and female infected patients (Figure 7, A and B). In the Mick data set (25), 13 genes presented different expression levels between these groups of infected patients (Figure 7C). Additionally, the Mick and Liberman data sets presented a considerable overlap of DEGs and shared functional enrichment categories. For instance, infected female patients showed reduced levels of CXCL8 receptors (*CXCR1* and *CXCR2*) in both swab data sets (Figure 7, A–D). We found that the downregulated genes in female patients who tested positive for SC2 were also enriched in the CMSP and NMI pathways (Supplemental Figure 6). These shared genes are essential for conventional neutrophil chemotaxis and the recruitment of polymorphonuclear neutrophil–MDSCs (33). Female patients also exhibited reduced levels of *IL-1β* when compared with male patients, a proinflammatory cytokine considered a potential target for COVID-19 therapy (34). Other proinflammatory transcripts (neurobeachin-like 2 [*NBEAL2*]) critical for leukocyte recruitment and granule exocytosis (35, 36) or alarmins that are released during tissue damage (S100 calcium binding protein A9 [*S100A9*]) (37, 38) were lower in swabs from female patients infected with SARS-CoV-2 in comparison with male patients. The differential expression analysis between female and male samples, both SARS-CoV-2–negative groups, showed downregulation of several genes belonging to the CMSP term (Supplemental Figure 7, A and B). Among them are promoters of the H_2_O_2_ production in airways (NADPH oxidase enzymes dual oxidase 2 [*DUOX2*]) (39), regulators of cell adhesion (fibronectin leucine-rich transmembrane protein 2 [*FLRT2*]), and proliferation (laminin B-2). In contrast, NMI genes were upregulated in Neg. SC2 females compared with Neg. SC2 males (Supplemental Figure 7, A–C). These results show that, even in the absence of the SARS-CoV-2, female subjects present a different expression pattern of these genes, indicating that the immune response may be sex dependent. This mucosal baseline state of reduced CMSP and increased NMI resembles an immune-protective quiescent-like (40–42) state. Beyond the genes mentioned above, female patients with COVID-19 presented 7 exclusive DEGs compared with the other SARS-CoV-2–positive groups (Supplemental Table 14). This differential expression reinforces the possibility of a better-modulated transcriptome profile of female subjects in response to SARS-CoV-2.

To further investigate the behavior of genes differentially expressed in female subjects with SC2 (Figure 7, A and B), we performed a multivariate regression (MVR) analysis under the assumption of normality distribution of the residuals. This statistical approach indicated that female subjects tend to systemically have a subtle lower gene expression pattern of some NMI and CMSP genes compared with male subjects infected with SARS-CoV-2 (Figure 7E and Supplemental Figure 8, A and B). Here, we controlled the effects of age, viral load, and composite expression of the other CMSP and NMI genes (Supplemental Figure 9). Likewise, the MVR of the expression of these DEGs suggests that not all of them are significantly affected by viral load or age. This indicates that the modulation of gene expression associates exclusively with sex differences (Figure 7E and Supplemental Figure 8). Given that *CXCR6* was recently associated with severe COVID-19 presenting with respiratory failure (43), we also analyzed its correlation with sex and viral load. There were no sex differences (logFC 0.299 and adjusted *P* = 0.9085; data not shown) in terms of gene expression levels. However, the expression of *CXCR6* between male and female patients was dependent on viral load, i.e., *CXCR6* behaves in opposite directions in male and female subjects, as assessed through a fully distributional regression using a GAMLSS model (Supplemental Figure 10).

**Figure 8:**
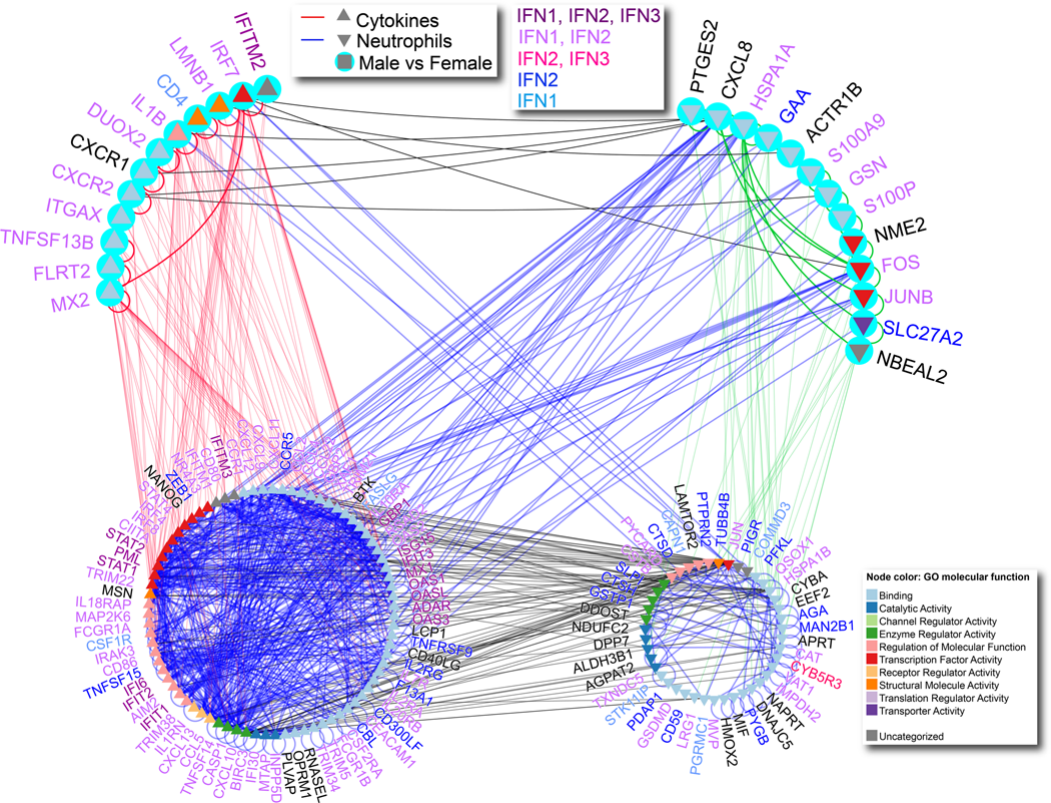
Network of cytokine-mediated signaling pathway and neutrophil-mediated immunity. Interactome between cytokine-mediated signaling pathway (CMSP) and neutrophil-mediated immunity (NMI) genes, highlighting differentially expressed genes (DEGs) in female samples. Node color represents the GO molecular function associated with DEGs. Triangles pointing up indicate CMSP genes, and triangles pointing down indicate NMI genes. The light blue circles represent DEGs in both infected and uninfected females. Blue edges highlight the NMI interactions; red edges reflect the CMSP interactions. The lower left subnetwork and lower right subnetwork show the interactions between CMSP and NMI genes, respectively. The label color represents the type of interferome associated with the gene. The interaction network was visualized using NAViGaTOR. GO, gene ontology.

Finally, to better understand this sex dimorphism in the relationship of CMSP and NMI genes in COVID-19, we performed an interferome analysis of these 2 sets of DEGs. This analysis revealed an interconnected network of IFN-regulated genes modulated by IFN type I, II, and III (Figure 8 and Supplemental Tables 15 and 16).

## Discussion

Here, we employed a systems and integrative immunology investigation aiming to identify transcriptome changes to gain novel insights into the higher male/female mortality ratio observed in patients with COVID-19. We consistently found several upregulated CMSP and downregulated NMI DEGs throughout different public data sets of large cohorts of patients infected with SARS-CoV-2 (swabs from GSE152075 and GSE156063 and leukocytes from GSE157103) (24, 25, 30). CCA and interferome analysis indicated that these DEGs form an interconnected network of several IFN-regulated genes. Our approach suggests that female and young patients exhibit a higher transcriptional modulation capacity than male and elderly patients. In support of this observation, female and young patients have a stronger downregulation of the expression of inflammatory genes, such as CXCL8 receptors (*CXCR1* and *CXCR2*), *IL-1β*, *NBEAL2*, and *S100A9*, when compared with male and elderly subjects. These genes are considered key players in immunological pathways involved in multiorgan injury and consequent death reported in COVID-19 (16, 17, 33, 44–46). For instance, genetic deletion or therapeutic inhibition of CXCR2 has been reported to improve the course of many inflammatory diseases (47). Likewise, the IL-1β blockade by anakinra improves COVID-19 outcomes in 72% of patients (48). This supports our findings suggesting that DEGs of females compared with males might be new candidates for COVID-19 target therapy. Moreover, our work is in line with a recent report by Takahashi et al. showing lower plasma levels of *CXCL8* in female than male patients (9). Therefore, females may have protective transcriptional plasticity against harmful inflammation and the consequent tissue damage caused by the SARS-CoV-2 infection.

The role of neutrophils in the pathophysiology of COVID-19 is currently debated. While these cells are classically known to play an essential role in the immune response against bacterial and fungal infections, their antiviral function has only recently been characterized (49–51). Neutrophils infiltrate the respiratory tract during viral infection and are required for a protective immune response against coronavirus. They also significantly contribute to respiratory tract pathology, i.e., hemorrhagic lesions, epithelial barrier permeability, and cellular inflammation in the lungs (52). Neutrophilia has consistently been found in patients with COVID-19 and correlates with worse clinical outcomes (14, 53–55). However, recent studies assessing the activation status of neutrophils in patients with COVID-19 have come to somewhat paradoxical results. Some investigators have identified that severe SARS-CoV-2 infection is associated with excessive release of reactive oxygen species and NETs (14, 15). Conversely, other studies demonstrated that the expansion of MDSCs increased with the severity of COVID-19, which agrees with our report of downregulated NMI signature (16, 18, 56).

MDSCs are a heterogeneous group of immature myeloid cells that can nonspecifically suppress T and B lymphocyte responses (18, 23). Much of our knowledge about MDSCs has been obtained through cancer studies (56), showing that tumors manipulate the myeloid system to evade the host immune response (22). However, the physiological role of MDSCs has become increasingly evident in COVID-19. The results of several studies suggest that MDSCs are components of the healthy immune system and play a protective role in homeostatic and disease contexts. MDSCs expand when necessary to protect the host against tissue damage during autoimmune and inflammatory diseases (19, 20), traumatic stress, transplantation, and sepsis (21–23). While MDSCs may potentially represent biomarkers of COVID-19 severity, their presence suggests an attempt of the host to modulate the severe immune dysregulation triggered by SARS-CoV-2. Notably, experimental models have shown that MDSCs from females, but not males, have a protective role in virally induced harmful inflammation that often causes sexually dimorphic myocarditis with increased incidence and mortality in males. Likewise, the high capacity of women to better modulate CMSP and NMI genes suggests a protective mechanism against severe COVID-19.

Notably, hyperinduction of IFNs following systemic activation of IFN-related genes has life-threatening immunopathological effects in COVID-19, despite playing a central role in antiviral immunity (44). The interferome network, when well orchestrated, is protective not only by promoting an antiviral milieu but also by limiting airway inflammation by directly modulating pathogenic neutrophil accumulation (57). Therefore, to address the threshold that shifts the IFN milieu from a protective to detrimental state will be imperative in identifying markers that allow for appropriate therapeutic administration of IFN or JAK inhibitors to selected patients with COVID-19 (58, 59).

While our study offers a possible explanation for the differences between mortality and morbidity between males and females with COVID-19, further potential mechanisms should be addressed. For instance, it remains to be determined if the sex dimorphic modulation of CMSP and NMI genes is specific for COVID-19 or if this occurs in other viral infections. Furthermore, the sex-driven dimorphic immune response could be due to the location of several immune genes or immune regulatory genes on the X chromosome (60) or related to estrogen receptor signaling, which is protective for SARS-CoV (60). Moreover, future studies need to evaluate the possible effect of sex-related risk factors for mortality in COVID-19 (smoking, alcohol consumption, and comorbidities) (61–63) on the immune response against SARS-CoV-2.

The SARS-CoV-2 infects and replicates in both upper and lower respiratory tracts (64). In this area, control of viral spread depends on interactions between epithelial and immune cells, which are mediated by cytokine signaling and cell contact (65). Here, we also found reduced expression of epithelial cell markers, such as the forkhead box protein J1 (*FOXJ1*), a master transcription factor for ciliated cells formation and function (66), and the secretoglobin family 1A member 1 (*SCGB1A1*), a defense protein highly secreted by Clara cells present in the airway epithelium (67) (Supplemental Figure 11). However, these genes were not differentially expressed among female versus male patients, suggesting that this phenomenon is not involved in the sex dimorphism associated with COVID-19. These data indicate that the SARS-CoV-2 infection in upper airways leads to tissue damage during the inflammatory response, as previously reported (64), with a possible broad loss of normal defensive protein secretion in the airways (68). Therefore, the landscape of differentially expressed immunological genes that we are reporting can likely not be explained by an altered influx of immune cells alone. It is possible that several events might be involved in the up- and downregulation of CMSP and NMI genes, such as disruption of interactions between epithelial and immune cells (64). However, these are limitations that need to be addressed by future histopathological studies.

In conclusion, our systemic and integrative approach suggests that the gene expression profile associated with CMSP and NMI has a distinct pattern according to sex and age in patients with COVID-19. This indicates the existence of specific immune-regulatory pathways underlying the sexual dimorphism of COVID-19 morbidity and mortality. This transcriptomic profile reveals pathways for the development of targeted therapies to improve the outcomes of COVID-19.

## Methods

### Data collection and differential expression analysis

Lieberman et al. (24) and Mick et al. (25) recently performed RNA-Seq experiments investigating global transcriptional profiles of nasopharyngeal swabs from 668 SC2 and 157 Neg. SC2) (data accessible at NCBI Gene Expression Omnibus [GEO] database (69), accession GSE152075 (24) and GSE156063 (25)). We retrieved the data sets to characterize the immunological signature. Read counts were transformed (log_2_ count per million [CPM]) and differentially expressed transcripts between groups were identified through the web tool NetworkAnalyst 3.0 (https://www.networkanalyst.ca/) (70) using the limma-voom pipeline (71). Age (young individuals, <60 years; elderly individuals, ≥60), sex (male and female), and viral load (only for GSE152075) were categorized as previously described (24). As recommended by FDA (https://www.fda.gov/media/136873/download), the viral load was classified according to an RT-qPCR test. This method evaluates the cycle threshold (Ct) of the SARS-CoV-2 nucleocapsid gene region 1 (N1) target during diagnostic RT-qPCR and assumes low viral load when N1 Ct >24 and high viral load if N1 Ct <19. We applied the statistical cut-offs of log_2_ fold change >1 and adjusted *P* < 0.05 to determine DEGs between the categories. For subsequent analysis, we followed the limma-voom pipeline to identify DEGs between female and male patients.

### Enrichment analysis and data visualization

We used these DEGs to identify different ontology terms. Biological processes (GO) were analyzed using EnrichR (http://amp.pharm.mssm.edu/Enrichr/) (26, 27), and the enriched immunological terms were generated according to adjusted *P* < 0.05 and *Z*-score (correction to the test) in a combined score provided by EnrichR database (26, 27). The biological process terms were included in an integrative analysis using the criterion of overrepresentation (log_2_ combined score >2) in at least 2 categories. Concomitantly, we performed the analysis of gene coexpression modules with the R package CEMiTool using default parameters (28). We plotted the set of genes associated with CMSP (GO:0019221) and NMI (GO:0002446) in bubble-based heat maps with hierarchical clustering using the web tool Morpheus (https://software.broadinstitute.org/morpheus/) (72) with Euclidian distance metric. GraphPad Prisma v.8 was used to generate the violin plots. The UniProtKB database (http://www.uniprot.org/) was used to access the functional information. We used clusterProfiler (73) to obtain dot plots and Cnetplots of enriched terms associated with CMSP- and NMI-associated genes. Shared CMSP and NMI genes among all groups were displayed using Circos plot (http://circos.ca/) (74). The identification of interferome genes was performed with Interferome v2.01 (http://www.interferome.org/interferome/home.jspx) (32).

### Molecular network of CMSP and NMI genes

The network of CMSP and NMI was constricted using DEGs in COVID-19, and we highlighted the genes differentially expressed in female samples. DEGs were used as input into Integrated Interactions Database (IID version 2020-05; http://ophid.utoronto.ca/iid) (75, 76) to identify direct physical protein interactions. The resulting network was annotated, analyzed, and visualized using NAViGaTOR 3.013 (77). The final network was exported in SVG format and finalized with legends in Adobe Illustrator. In Figure 8, node color represents GO molecular function as per the legend. Triangles pointing up indicate CMSP genes, and triangles pointing down indicate NMI genes. The light blue circles represent DEGs in females in both infected and uninfected samples. Blue edges highlight NMI interactions, and red edges reflect CMSP interactions. The lower left subnetwork and lower right subnetwork show the interactions between CMSP and NMI genes, respectively. Protein name color represents the type of interferome associated with the gene.

### Code availability

Analysis notebooks are available at https://github.com/DesireePlaca/FREIREPP_COVID19_MS1.git

### Data availability

Publicly RNA-Seq data sets analyzed herein are available at GEO, under accession numbers GSE152075, GSE157103, and GSE156063. Single-cell RNA-Seq data (data set EGA00001004571) was obtained from neutrophil clusters, as reported by Schulte-Schrepping et al. (16).

### Statistics

Before the application of the statistical methods described below, the variable transformation was performed as described in each figure legend. For gene expression data, we added unity to all counts and consecutively applied a base 2 logarithmic function for each gene variable, herein called transformed gene expression. The remaining quantitative variables were scaled. Only CMSP and NMI genes were included in the data analysis as response variables. We used NP-MANOVA (31) to test differences in the mean vectors of gene expressions between SC2 and Neg. SC2 groups separately for both CMSP and NMI genes. *P* values of less than 0.05 were considered significant.

#### Relative effect analysis.

For each gene, relative effects (31) of transformed expression were compared between SC2 and Neg. SC2 groups, with associated 95% confidence intervals, calculated via bootstrap simulation using the method of resampling pairs (78). Bootstrap statistics were based on 1000 simulations and percentile confidence intervals (78).

#### CCA.

CCA (79) was applied to investigate patterns of association between genes related to CMSP and NMI genes, considering the observations from the SC2 group alone. We retained the first two canonical variates for subsequent interpretations. Signed canonical correlations were calculated according to Jendoubi et al. (79).

#### Ridge regression.

We used ridge regression (80) for setting up a predictive model for the response variable viral load as a function of the regression covariates age, sex, and transformed gene expressions, considering observations from the SC2 group alone. Model estimates were obtained using 10-fold cross validation (80). As a testing set, 25% of the data set was kept and used for evaluating the model prediction accuracy.

#### MVR.

We performed MVR analysis with normally distributed additive errors (81) to model the mean vector of transformed gene expression associated with the 6 genes found with differentially expression in female patients (*CD14*, *CXCR1*, *CXCR2*, *IL1B*, *NBEAL2*, and *S100A9*), considering the observations from the SC2 group alone. The regression covariates included were viral load and the principal component scores of the remaining genes as well as their interaction with sex. The variable sex was coded using 1 for male and 0 for female. Principal component scores resulted from a principal component analysis (PCA) based on the transformed gene expressions for genes not included as responses in the MVR mode. After PCA, the estimated matrix of loadings was rotated using the varimax criterion. The likelihood ratio and Wald statistics were used, respectively, for testing the generalized hypothesis involving MRV model parameters and obtaining the statistical significance of each regression coefficient individually (81). The level of significance for all hypothesis testing was fixed at 5%, and the wild bootstrap method was used for calculating *P* values and parameter standard errors robust against heteroscedasticity on the regression errors (82). Bootstrap statistics were based on 1000 simulations (78). Model adequacy was studied using the metrics developed in Díaz-García et al. (81).

#### GAMLSS regression.

The expression of *CXCR6* was modeled using the framework of the generalized additive models for location, scale, and shape (GAMLSS), under the assumption of a log-normal distribution adjusted for zeros for the response variable. The mean of the distribution and the log odds of 0 was modeled using as covariates age, viral load, and principal component scores for NMI and GMSP genes (PC1), as described above for MVR (see *MVR*) and their interaction with sex (where male was coded as 1 and female as 0).

#### Statistical software and packages.

The sample median and sample interquartile range were calculated using R software version 4.0.2 (https://www.r-project.org/index.html). The NP-MANOVA, CCA, MVR, and ridge regression analysis were all performed on the R software version 4.0.2. Specifically, we used the npmv package (31) for NP-MANOVA, the whitening (79), DFA, and CANCOR packages for CCA, the glmnet package for ridge regression, the psych package for PCA, and the GAMLSS package (83) for GAMLSS analysis of CXCR6 expression. The authors implemented MVR following the results from García et al. (2003) (81). Finally, all statistical graphs were constructed using the functionalities of the ggplot2 package (84).

### Study approval

As we used publicly available data, ethical approval was not applicable.

## Author contributions

PPF, AHCM, and OCM cowrote the manuscript and provided scientific insights. PPF, AHCM, DLMF, TDCH, IJ, and OCM performed bioinformatics analyses. PPF, GCB, RFC, LCSF, ACN, HIN, NOSC, VLGC, and OCM conceived and designed the study. PPF, AHCM, GCB, LFS, DLMF, RCS, ISF, SMSN, KTA, DRP, GCM, and OCM analyzed data. PPF, AHCM, GCB, LFS, NEK, LMG, GCM, RFC, LCSF, ACN, HIN, IJ, HDO, NOSC, VLGC, and OCM revised and edited the final manuscript. VLGC and OCM supervised the project.

## Supplementary Material

Supplemental data

Supplemental Tables 1-16

## Version 1 05/24/2021

Electronic publication
