# Supplementary material for: The relationship between cytokine and neutrophil gene network distinguishes SARS-CoV-2–infected patients by sex and age
Source: JCI Insight. 2021 May 24;6(10):e147535. doi: 10.1172/jci.insight.147535 (PMC8262322; doi:10.1172/jci.insight.147535)
Supplement: Supplemental data [file jciinsight-6-147535-s230.pdf]

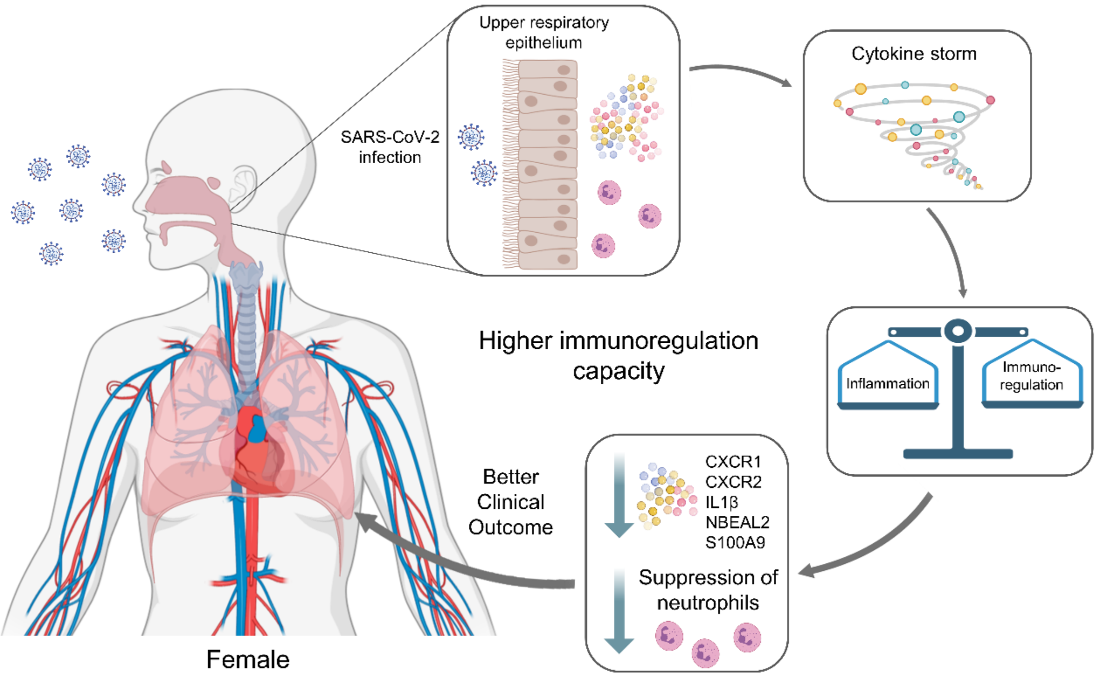

Graphical Abstract | Representative scheme of the female immunoregulation capacity after SC2 infection. Our results suggest that women can control the inflammation better than men due to a higher capacity to regulate the expression of genes encoding inflammatory molecules. We observed a decreased expression of chemoattractant receptors (*CXCR1* and *CXCR2*) and *IL1β* genes in women compared to men. This reduction might lead to lower neutrophil chemotaxis by CXCL8 and IL-1β-induced inflammation. Suppression of neutrophils could avoid tissue damage in the lung explaining the better clinical outcome observed among infected women. CXCR1: C-X-C Motif Chemokine Receptor 1; CXCR2: C-X-C Motif Chemokine Receptor 2; IL1β: Interleukin 1 beta; NBEAL2: Neurobeachin Like 2; S100A9: S100 Calcium Binding Protein A9. Credit: Created with BioRender (https://biorender.com/).

A

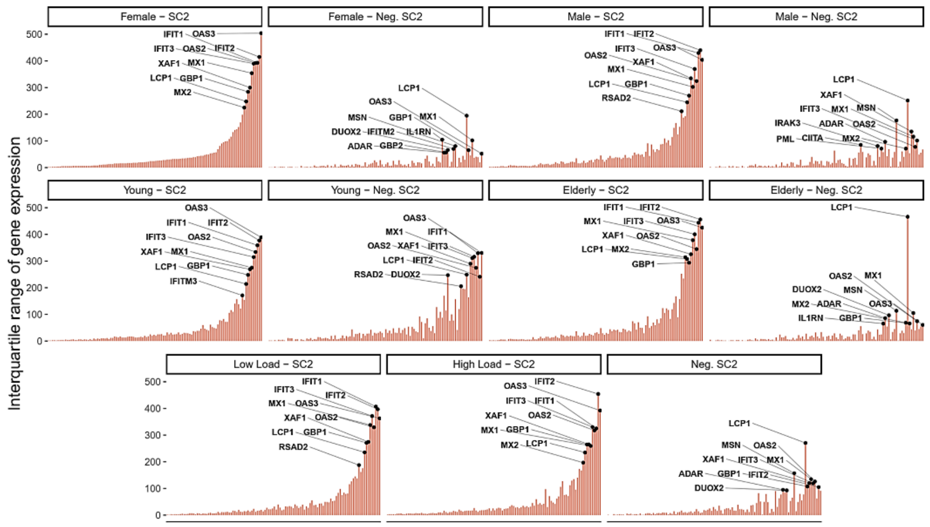

B

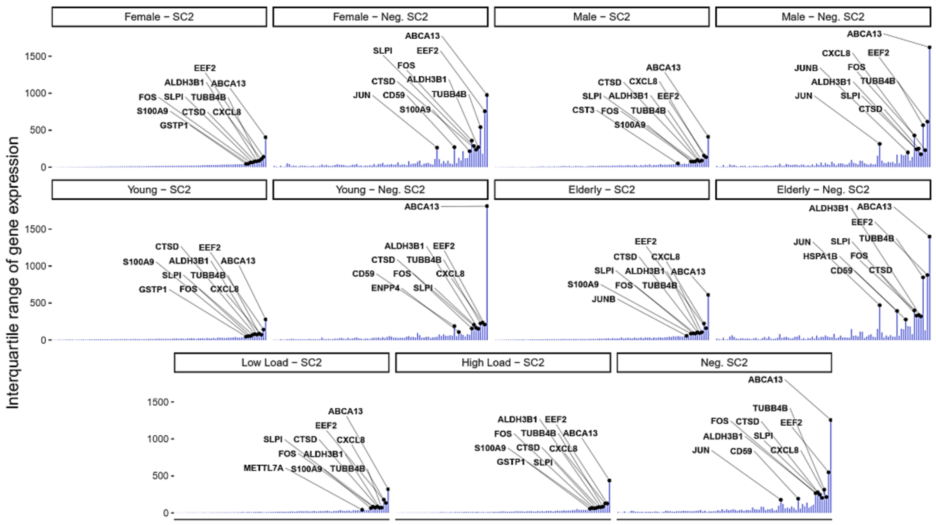

Extended Data Fig. 1 | Interquartile range of gene expression by gender, age, and viral load. A-B, Measure of variability (interquartile range) of gene expression of (**A**) cytokine-mediated signaling pathway (CMSP) and (**B**) neutrophil-mediated immunity (NMI) genes of viral load-, gender-, and age-matched SARS-CoV-2 (SC2) positive vs. negative (Neg. SC2) subjects.

A

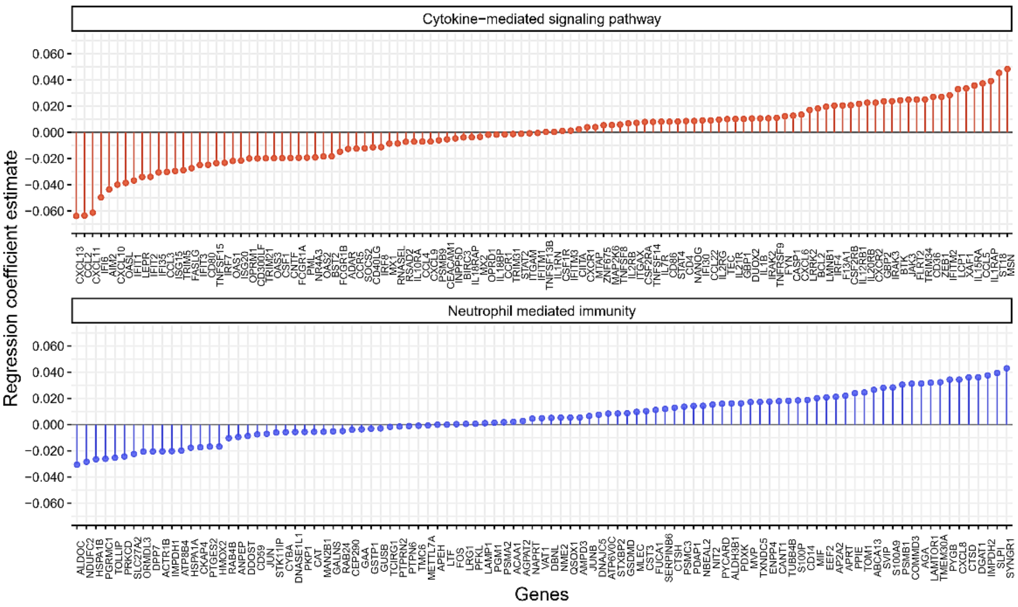

B

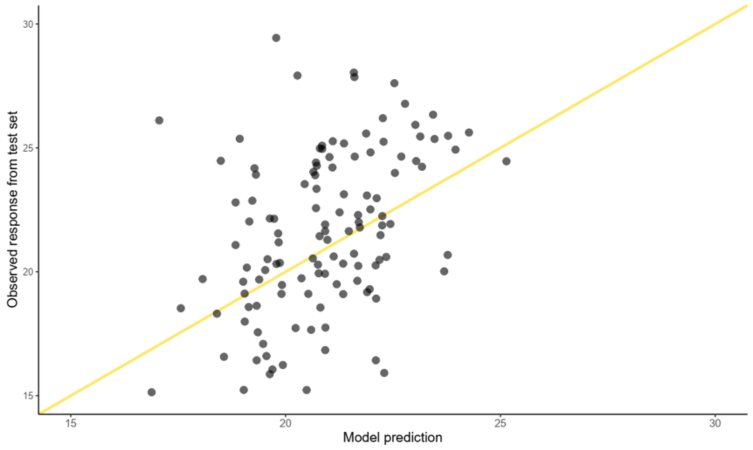

Extended Data Fig. 2 | The relationship between gene expression and viral load. A, The association between the expression of CMSP and NMI genes and viral load was calculated using Ridge regression. Only observations from the positive SC2 group were used for the estimation of parameters in the model. **B**, Accuracy of the ridge regression model fitted for estimating viral load as a linear function of age, gender, and transformed gene expressions. Each dot corresponds to an individual observation of the testing dataset, composed of 25% of the sample (whole SC2 positive group). The yellow line marks the bisection of the first quadrat in the cartesian plane and represents the situation where the model perfectly predicts the viral load for the test point. CMSP: cytokine-mediated signaling pathway, NMI: neutrophil mediated-immunity. SC2 = SARS-CoV-2 positive.

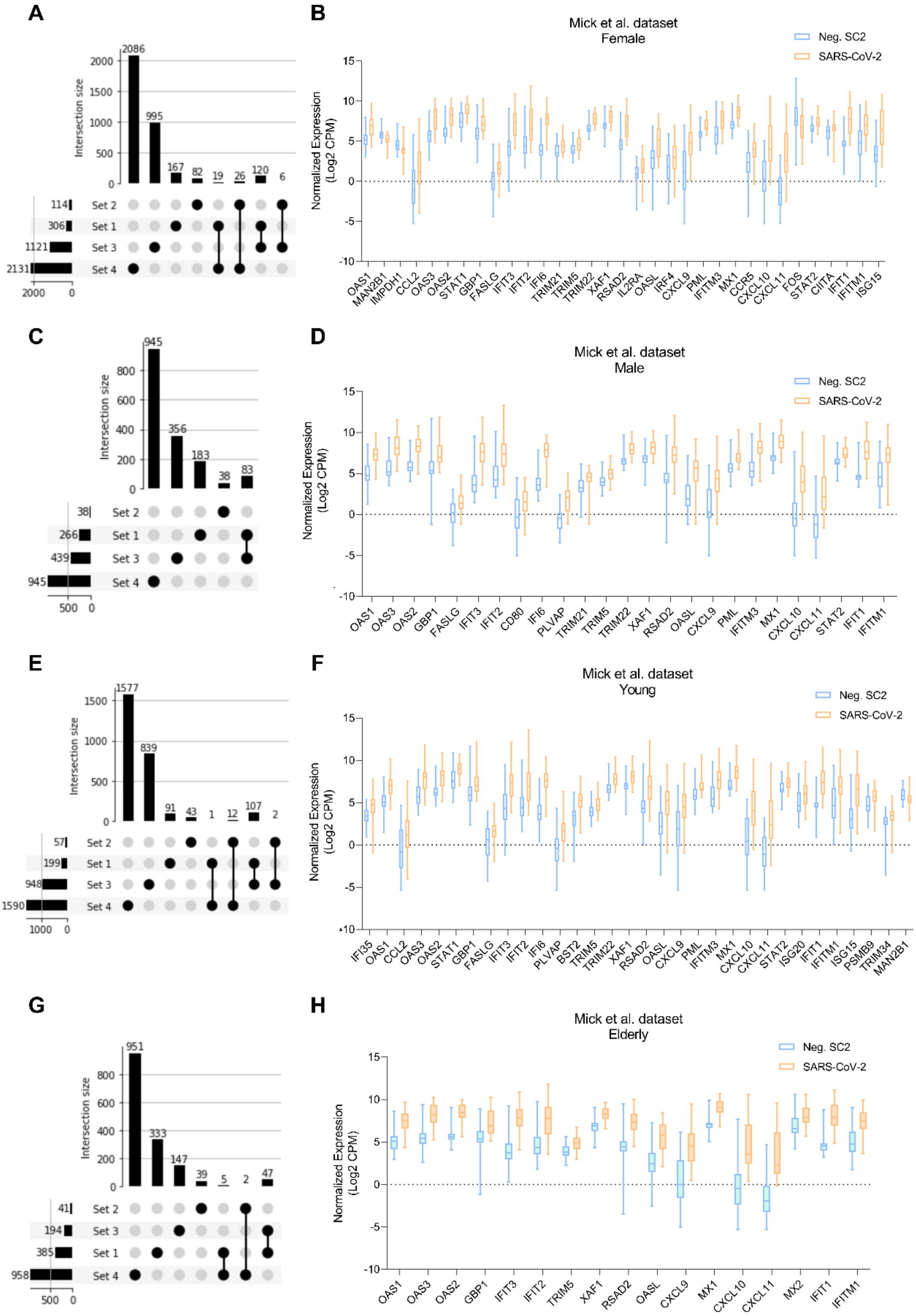

Extended Data Fig. 3 | Reproducibility of cytokine-mediated signaling pathway and neutrophil-mediated immunity genes in swab samples (GSE156063). A, **C**, **E**, **G**, UpSet plots displaying the set intersections for upregulated and downregulated genes in the Lieberman *et al*. dataset (GSE152075) and Mick *et al*. dataset (GSE156063) female samples (**A**), male (**C**), young (**E**), and elderly (**G**). **B**, **D**, **F**, **H,** Boxplot showing the common differentially expressed genes in female (**B**), male (**D**), young (**F**), and elderly (**H**) samples from Mick et al. dataset. Data are presented as normalized expression (Log2 CPM) of SARS-

CoV-2 vs. negative SC2 samples, considering only the cytokine-mediated signaling pathway (CMSP) and neutrophil-mediated immunity (NMI) genes with adj *p*-value < 0.05 and Log2FC >1. Set 1: upregulated in Lieberman *et al*. dataset (GSE152075); Set 2: downregulated in Lieberman *et al*. dataset (GSE152075); Set 3: upregulated in Mick *et al*. dataset (GSE156063); Set 4: downregulated in Mick *et al*. dataset (GSE156063).

EXTENDED DATA FIG. 4

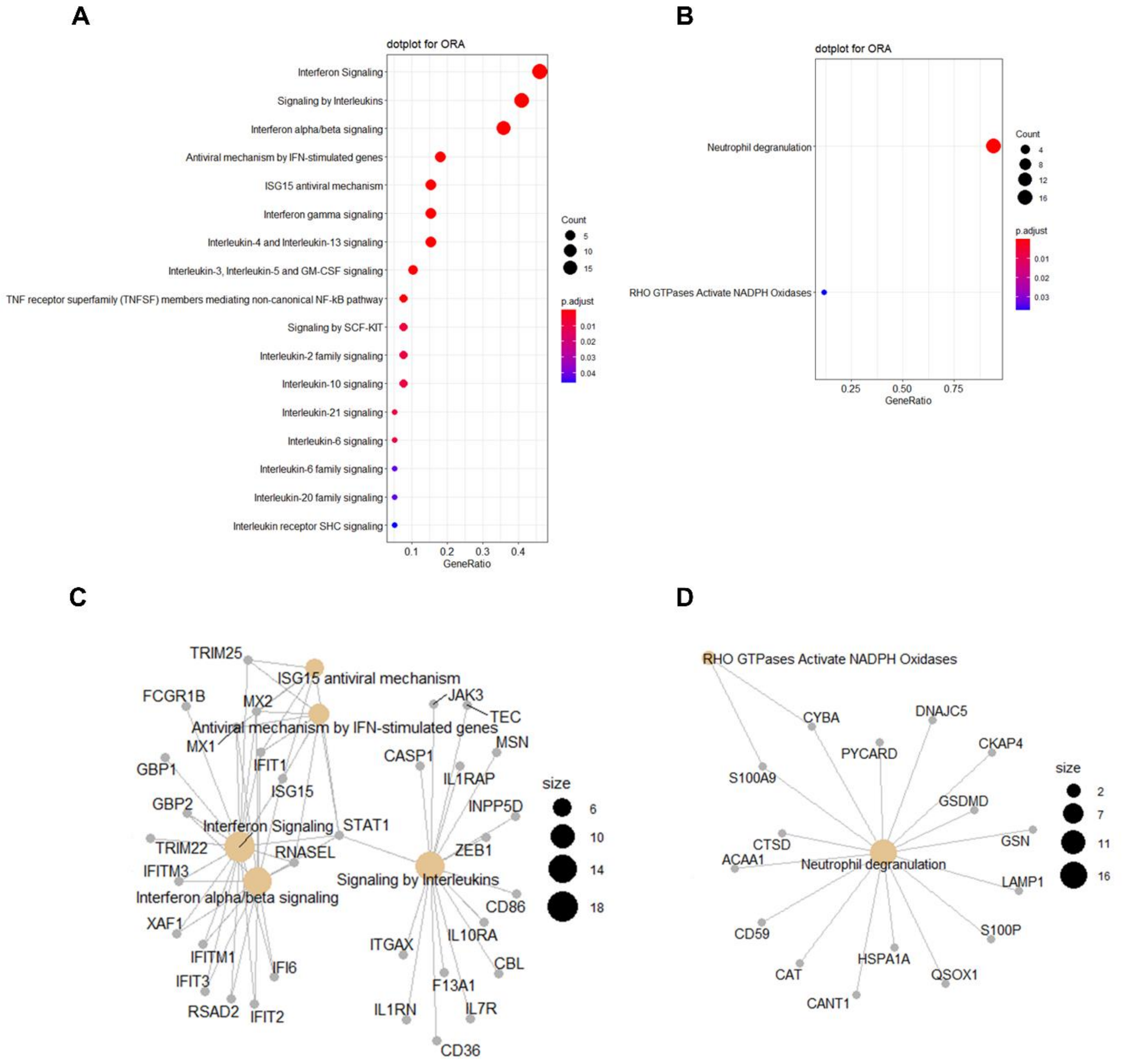

Extended Data Fig. 4 | Upregulated cytokine-mediated signaling pathway and downregulated neutrophil-mediated immunity genes identified by single-cell RNAseq (dataset EGAS00001004571). A-B, Dot plot of enriched terms **(A)** upregulated and (**B**) downregulated in neutrophil clusters obtained as described by Schulte-Schrepping et al.(16) **C, D,** Cnetplot of cytokine-mediated signaling pathway (CMSP) and neutrophil-mediated immunity (NMI)-associated genes. Graphics were generated using the ClusterProfiler package.

A

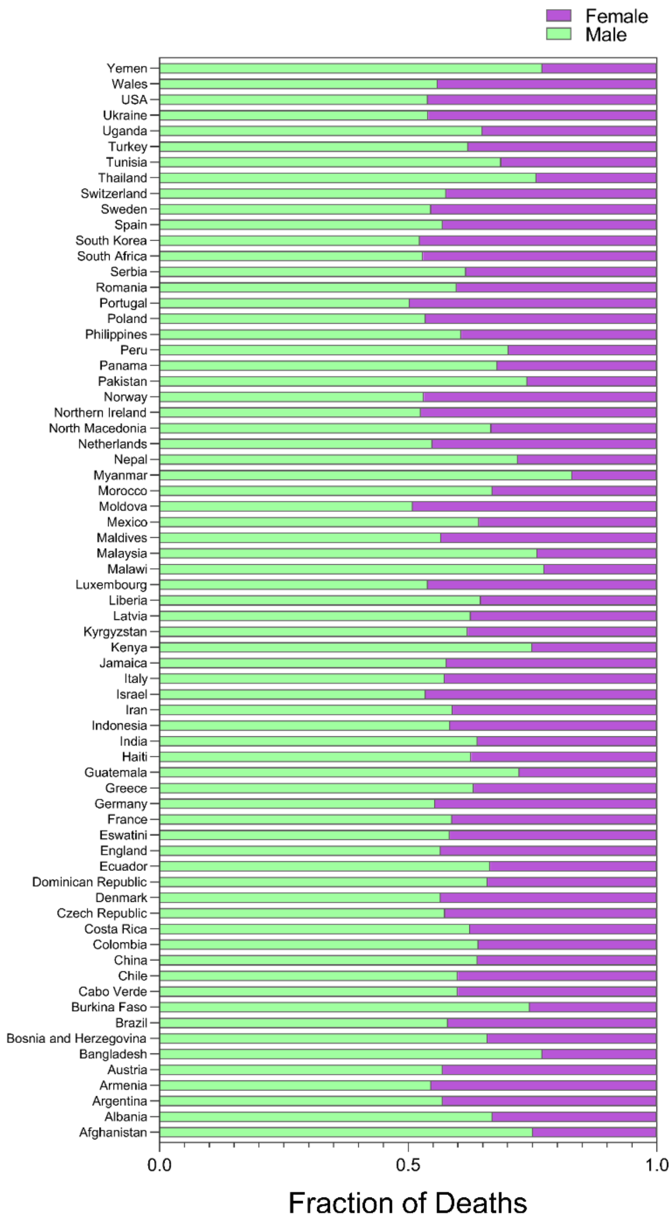

Extended Data Fig. 5 | Fraction of deaths due to COVID-19 is higher in males than in females. A, Fraction of male and female deaths by countries. Data were retrieved from https://globalhealth5050.org/the-sex-gender-and-covid-19-project/dataset/.

A

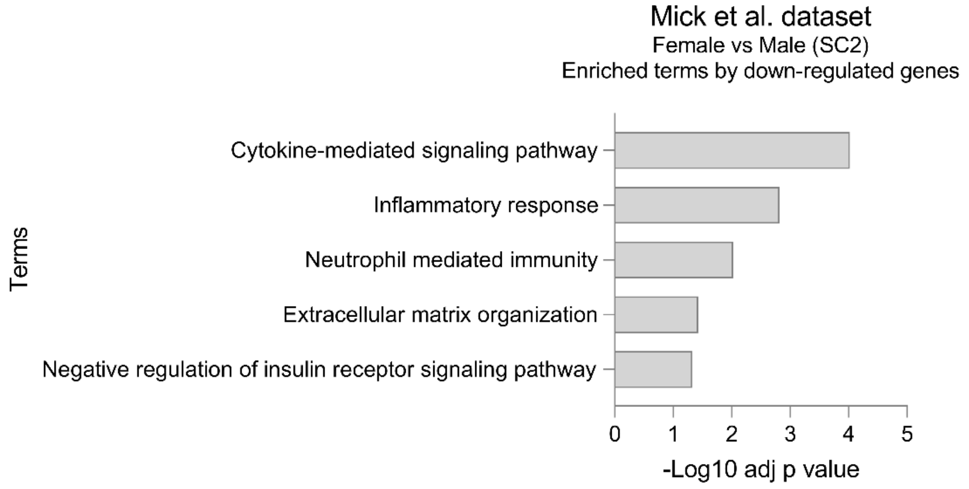

B

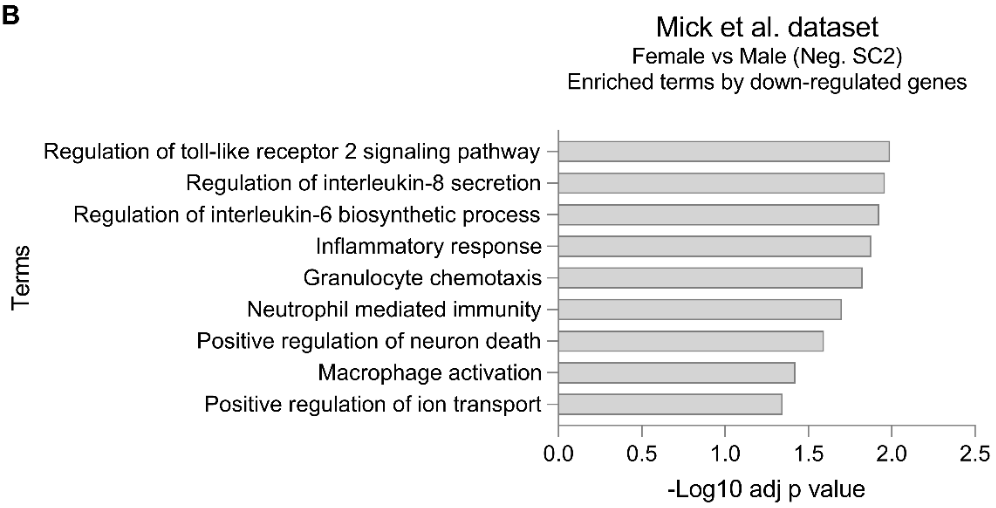

Extended Data Fig. 6 | Enrichment analysis of downregulated genes in female compared to male subjects from the Mick et al. dataset (GSE156063). A, Most enriched biological process terms (adj *p*-value< 0.05) by downregulated genes in female versus male subjects positive for SARS-CoV-2 (dataset GSE156063). **B**, most enriched biological process terms (adj *p*-value< 0.05) by downregulated genes in female versus male subjects negative for SARS-CoV-2 (dataset GSE156063).

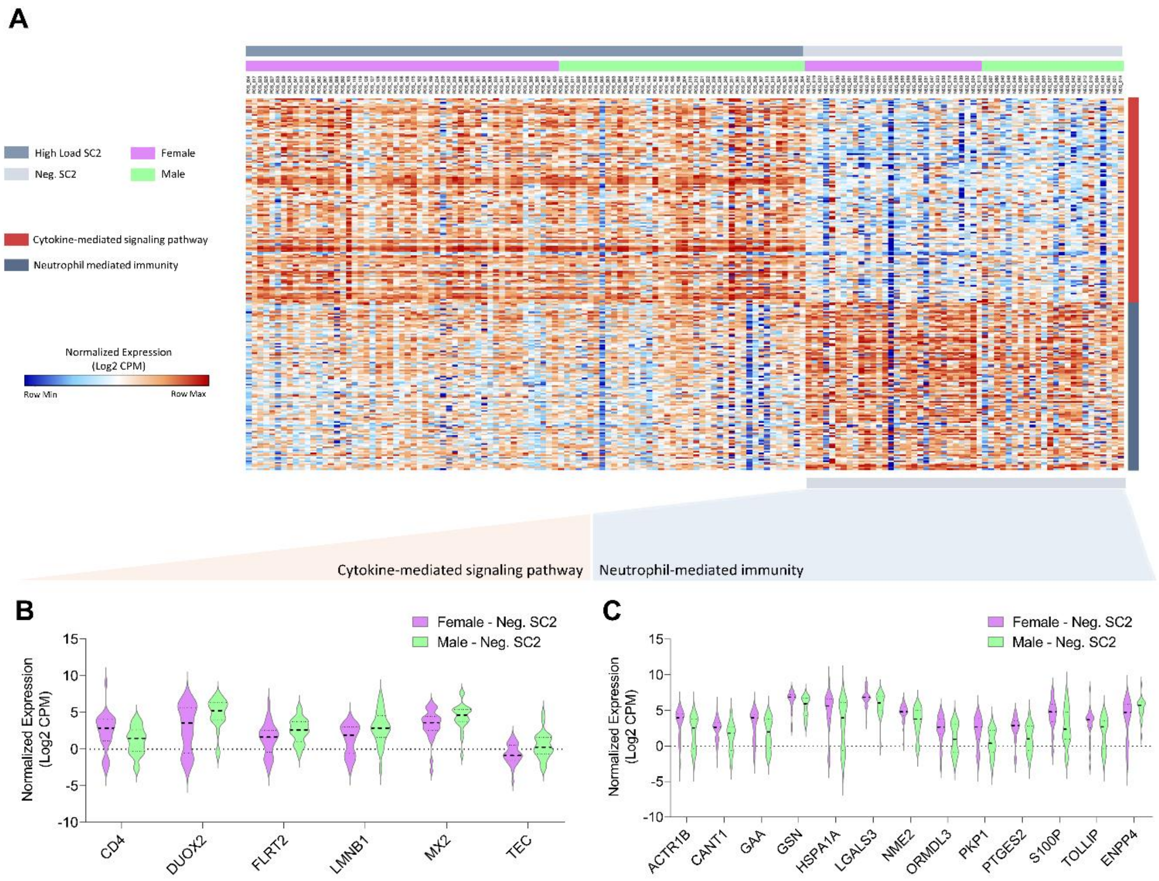

Extended Data Fig. 7 | Transcriptional differences in cytokine-mediated signaling pathway and neutrophil-mediated immunity distinguish females and males. A, Unsupervised hierarchical clustering heatmap of cytokine-mediated signaling pathway (CMSP) and neutrophil mediated immunity (NMI) genes (normalized gene expression in Log2 counts per million or CPM). On the right, red tracks denote CMSP genes, and blue tracks represent NMI genes. **B-C**, Violin plots present differentially expressed genes (DEGs, adjusted *p*-value < 0.05 and Log2 fold change (Log2FC) >1) of CMSP (**B**) and NMI (**C**) of negative SARS-CoV-2 (Neg. SC2) females versus males from the dataset GSE152075. All data of the violin plots are normalized expressions (Log2 CPM).

EXTENDED DATA FIG. 8

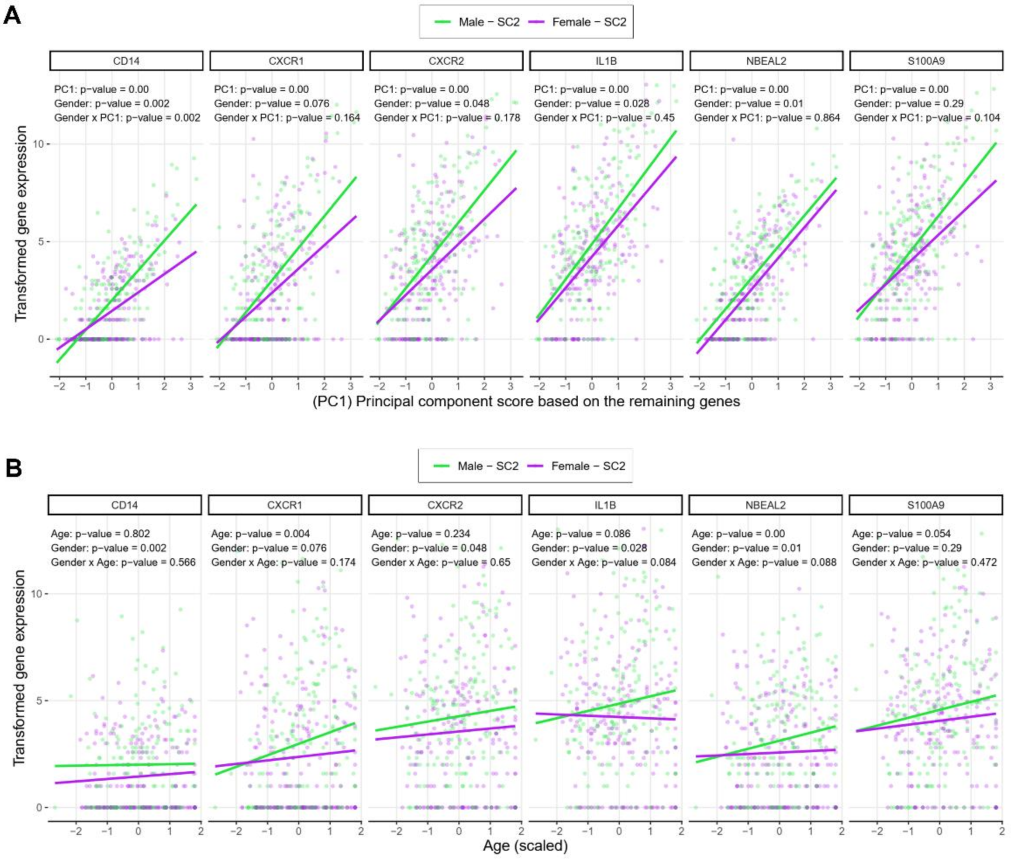

Extended Data Fig. 8 | Relationship between the gene expression with the principal component score and age. **A-B,** Multivariate regression of the transformed expression of six genes (*CD14*, *CXCR1*, *CXCR2*, *IL-1β*, *NBEAL2*, *S100A9*) on **A,** principal component score of the remaining genes (PC1) and **B,** Age. Each dot represents a unique individual sample (purple: female; green: male). PC1 or Age: *p*-value < 0.05 indicates a significant association of these variables with the expression of the corresponding gene among these six DEGs. Gender *p*-value ≤ 0.05 indicates significant differences in the mean expression of females compared to males regarding the corresponding gene among the six DEGs. Gender x PC1 or Gender Age: *p*-value ≤ 0.05 indicates significant changes between female and male along the gradient (continuum) of PC1 or age. The term *scaled* means that the variable was transformed by subtracting its mean and division by its standard deviation. Gene expression was transformed by adding unity and subsequently taking the base two logarithms. The variable gender was coded using 1 for male and 0 for female. PC1: Principal Component 1.

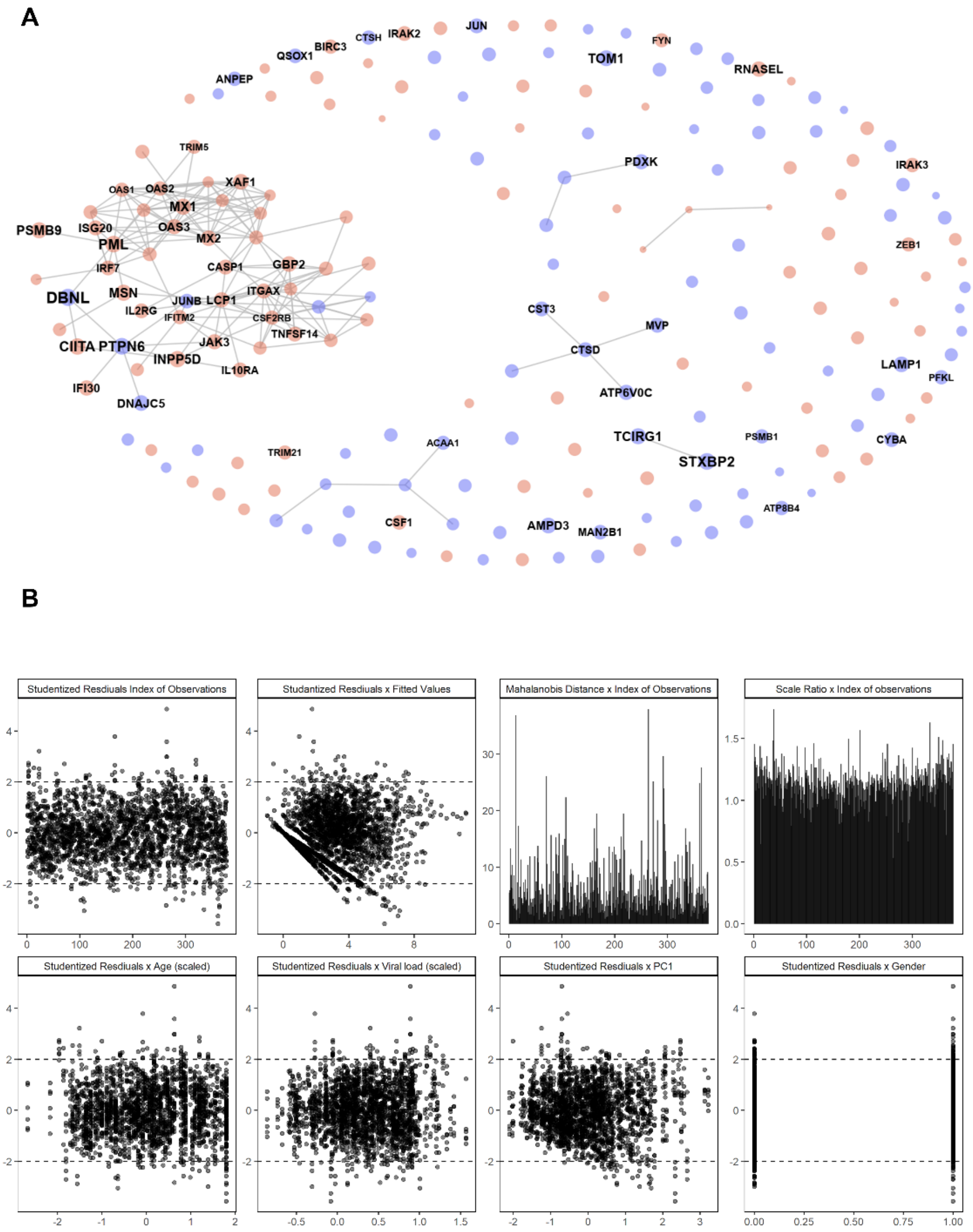

Extended Data Fig. 9 | Accessory graphs for the multivariate regression analysis. **A**, Graphical representation of the Pearson correlation matrix containing cytokine-mediated signaling pathway (CMSP) and neutrophil mediated immunity (NMI) genes, except *CD14*, *CXCR1*, *CXCR2*, *IL-1β*, *NBEAL2*, *S100A9.* Pairs of genes with a Pearson correlation coefficient ≥ 0.7 are connected by grey edges. Red and blue dots represent CMSP and NMI genes, respectively. Names are shown only for genes with loading estimate with absolute value ≥ 0.7 on the first principal component. The size of names and dots is proportional to the absolute value of the respective loading estimate. **B**, Panel showing several plots for analysis of residuals of the fitted multivariate regression model. To circumvent problems related to heteroscedasticity of the residuals, a wild bootstrap was used to estimate the p-values associated with all multivariate and univariate regression tests. The variable gender was coded using 1 for male and 0 for female.

A

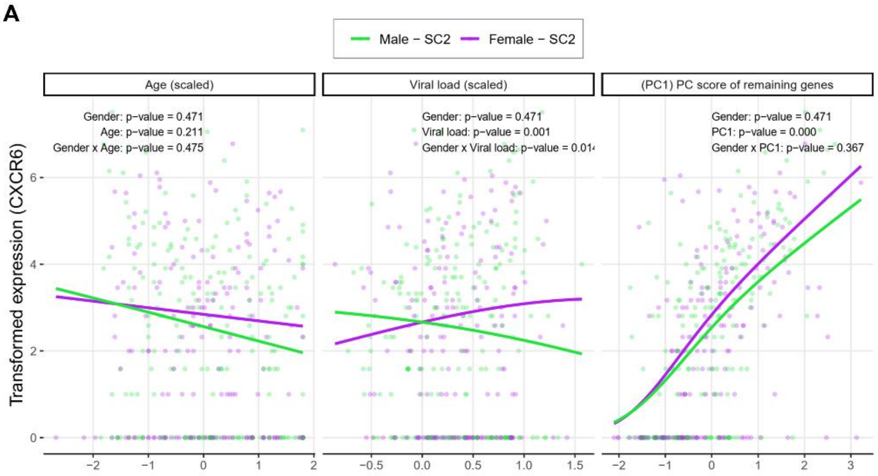

B

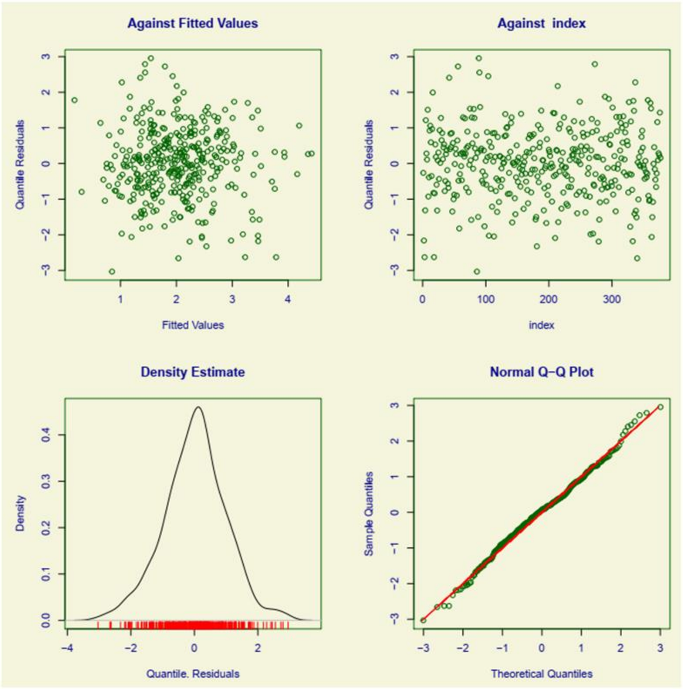

Extended Data Fig. 10 | Effect of viral load on the *CXCR6* expression. **A**, Regression analysis of *CXCR6* expression on age, viral load, principal component score (PC1) of remaining genes and their interaction with gender. For modeling, it was assumed a lognormal distribution adjusted for zeros (also known as the delta distribution) for the *CXCR6* expression. The mean and the log odds of a zero were modeled by linear predictors analogous to that used for the multivariate regression described in Statistical Methods Section. Gender *p*-value ≤ 0.05 indicates significant differences in the mean expression of *CXCR6* for females compared to males. Age, Viral load, or PC1: *p*-value ≤ 0.05 indicates a significant effect of the respective variable on *CXCR6* expression. Gender Age, Gender x Viral load or Gender x PC1: *p*-value ≤ 0.05 indicates significant changes between female and male along the gradient (continuum) of the respective variable. The term *scaled* means that the variable was transformed by subtracting its mean and division by its standard deviation. The variable gender was coded using 1 for male and 0 for female. **B**, Residual plots for the fitted GAMLSS, showing no critical deviations from the goodness of fit.

EXTENDED DATA FIG. 11

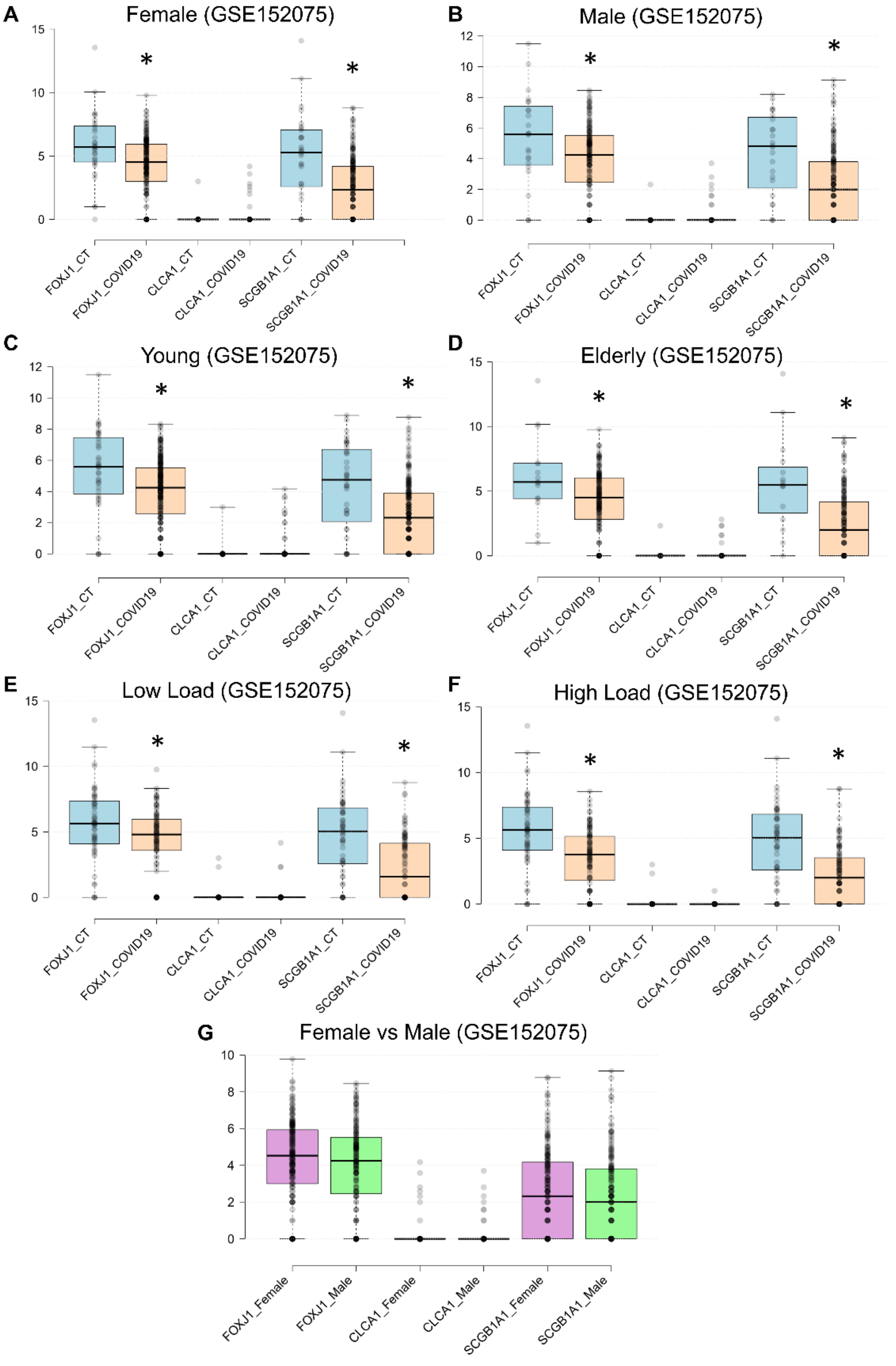

Extended Data Fig. 11 | Expression of epithelial cell markers *FOXJ1*, *CLCA1*, and *SCGB1A1* in swab samples. A-F, Boxplot showing the expression of *FOXJ1* (ciliated cells), *CLCA1* (goblet cells), and *SCGB1A1* (secretory cells) in female (**A**), male (**B**), young (**C**), elderly (**D**), low load (**E**), and high load samples (**F**). Data points show the normalized (Log2 counts) expression of genes from SARS-CoV-2 positive (COVID19) and negative (Control, CT) subjects. **G**, Normalized expression of *FOXJ1*, *CLCA1*, and *SCGB1A1* of infected females vs. males. *p < 0.05: statistical significance compared to the negative SC2 group.
